# Supplementary figures and images for: Structural analysis of rice Os4BGlu18 monolignol β-glucosidase
Source: PLoS One. 2021 Jan 20;16(1):e0241325. doi: 10.1371/journal.pone.0241325 (PMC7817009; doi:10.1371/journal.pone.0241325)

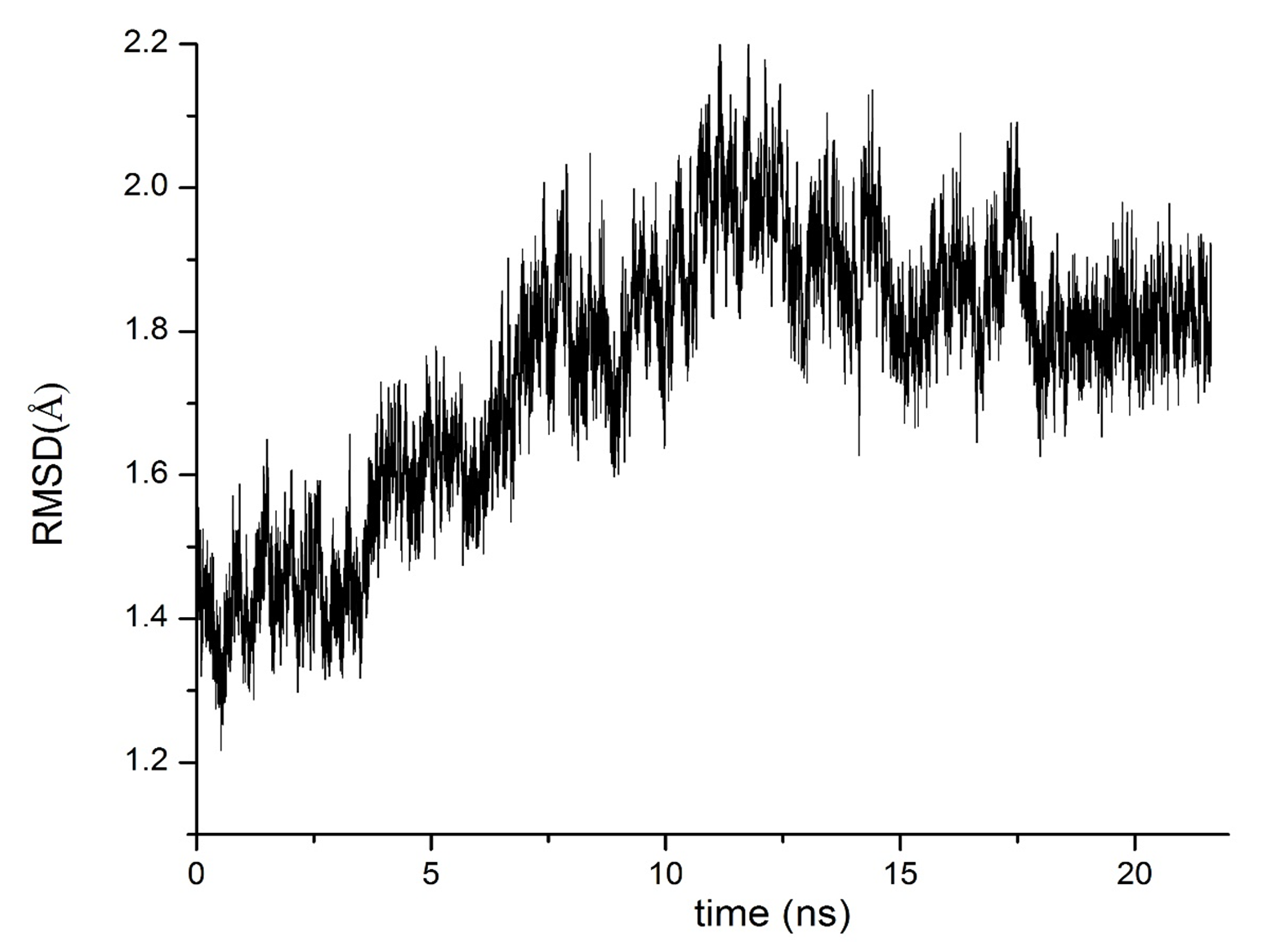

Supplement: S1 Fig — (TIF) [file pone.0241325.s001.tif]

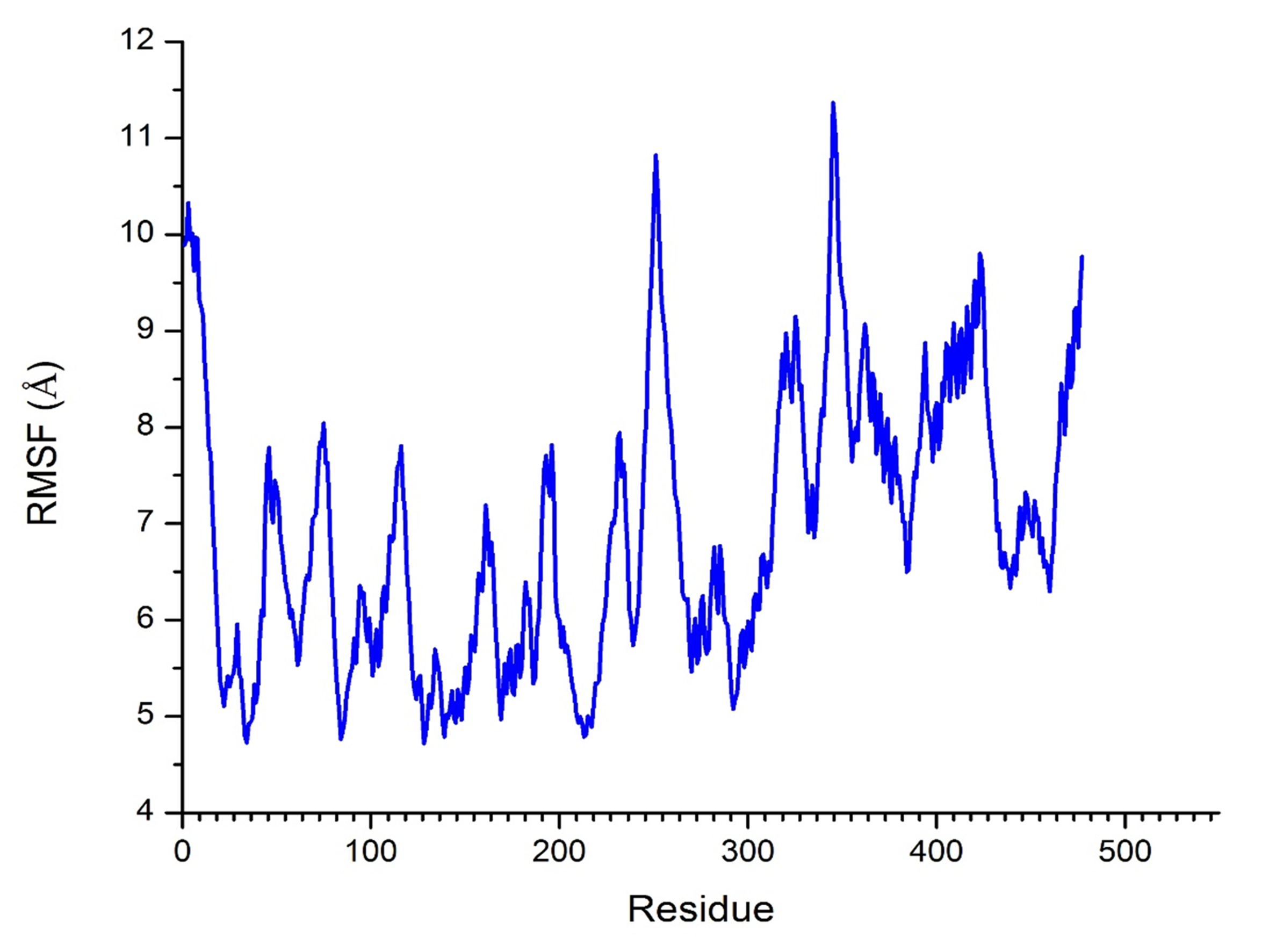

Supplement: S2 Fig — (TIF) [file pone.0241325.s002.tif]

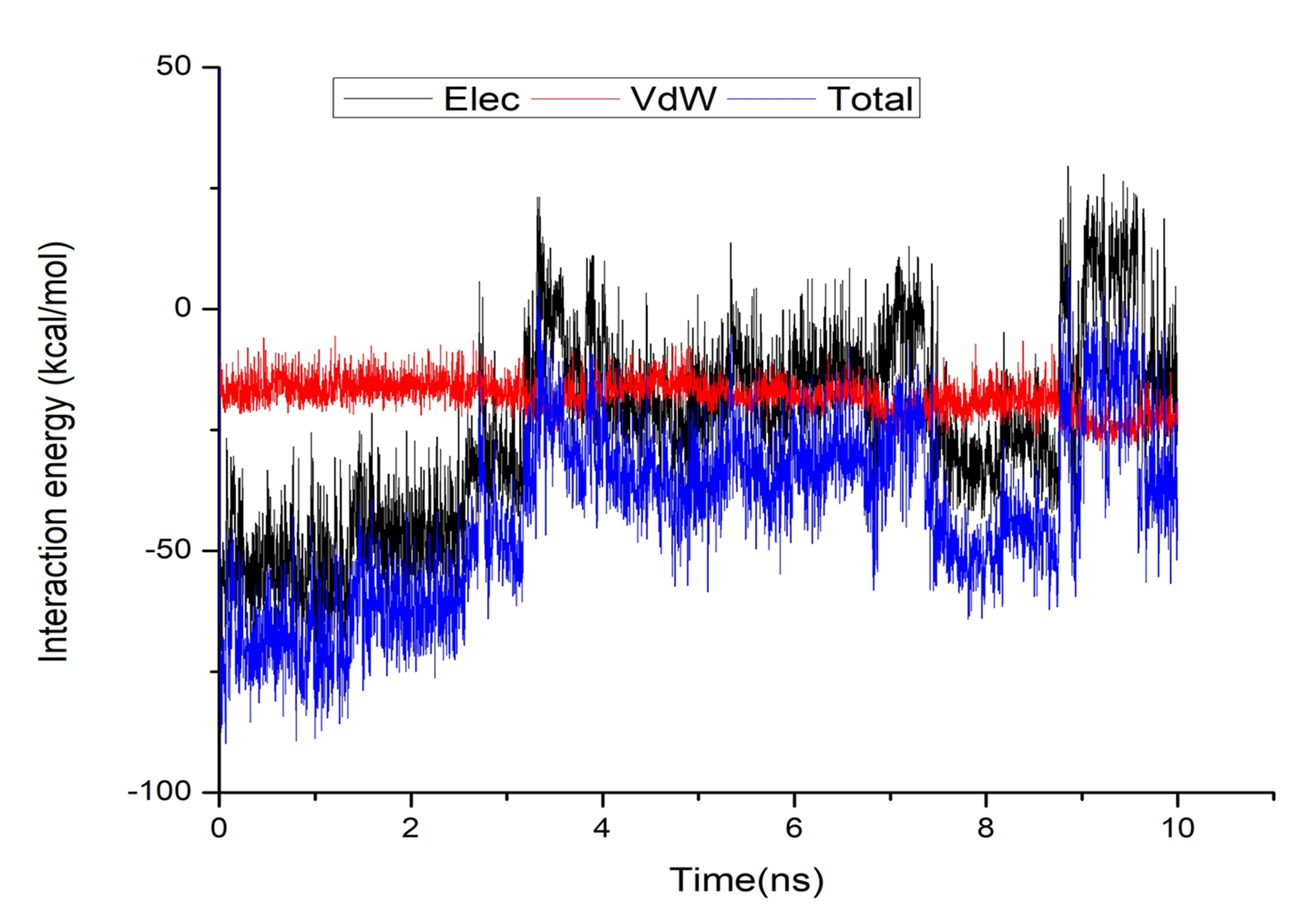

Supplement: S3 Fig — (TIF) [file pone.0241325.s003.tif]

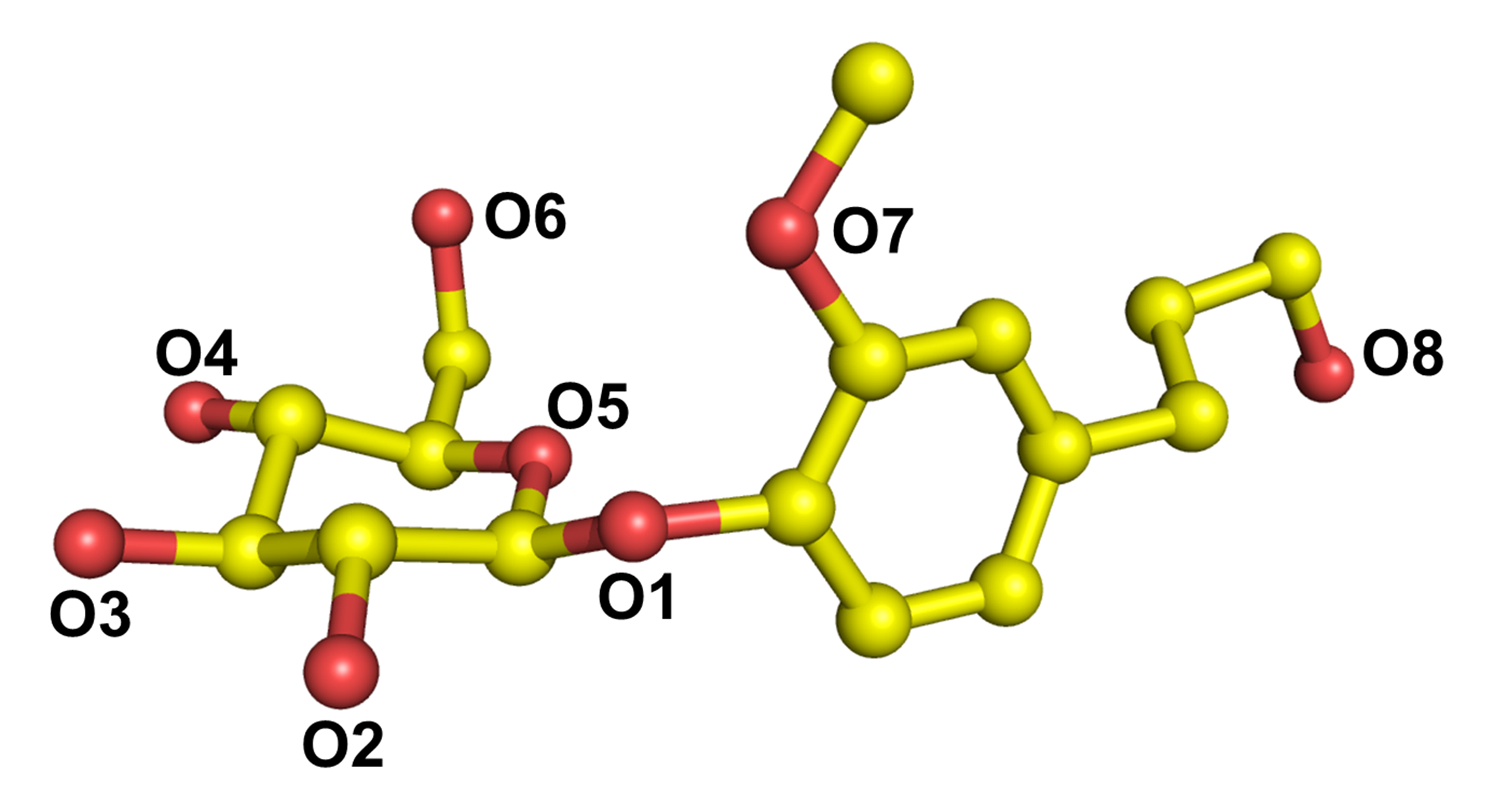

Supplement: S4 Fig — (TIF) [file pone.0241325.s004.tif]
