# Supplementary material for: Structural analysis of rice Os4BGlu18 monolignol β-glucosidase
Source: PLoS One. 2021 Jan 20;16(1):e0241325. doi: 10.1371/journal.pone.0241325 (PMC7817009; doi:10.1371/journal.pone.0241325)
Supplement: S1 Table — Hydrogen bond calculation was analyzed with a distance cutoff of 3.5 Å and an angle cutoff of 30°. Forty-six hydrogen bonds were found as listed. The numbering of the oxygen atoms is indicated in S4 Fig. (DOCX) [file pone.0241325.s005.docx]

**S1 Table. Hydrogen Bond Analysis.** Hydrogen bond calculation was analyzed with a distance cutoff of 3.5 Å and an angle cutoff of 30°. Forty-six hydrogen bonds were found as listed. The numbering of the oxygen atoms is indicated in Figure S4.

| Donor | Acceptor | %Occupancy |
| --- | --- | --- |
| \| CON1-Side-O2 \| \| --- \| \| CON1-Side-O3 \| \| TYR337-SideOH \| \| TRP465-Side-NE1 \| \| CON1-Side-O2 \| \| CON1-Side-O6 \| \| CON1-Side-O2 \| \| CON1-Side-O2 \| \| CON1-Side-O8 \| \| CON1-Side-O8 \| \| ASN193-Side-ND2 \| \| TYR208-Side-OH \| \| THR380-Side-OG1 \| \| CON1-Side-O5 \| \| CON1-Side-O6 \| \| TRP465-Side-NE1 \| \| TRP465-Side-NE1 \| \| HSE148-Side-NE2 \| \| TRP465-Side-NE1 \| \| CON1-Side-O3 \| \| CON1-Side-O4 \| \| CON1-Side-O1 \| \| TYR337-Side-OH \| \| CON1-Side-O3 \| \| TRP457-Side-NE1 \| \| CON1-Side-O2 \| \| CON1-Side-O4 \| \| CON1-Side-O6 \| \| HSE201-Side-NE2 \| \| CON1-Side-O2 \| \| CON1-Side-O3 \| \| HSE201-Side-NE2 \| \| CON1-Side-O8 \| \| CON1-Side-O8 \| \| CON1-Side-O3 \| \| CON1-Side-O1 \| \| TRP465-Side-NE1 \| \| CON1-Side-O4 \| \| TYR149-Side-OH \| \| TRP457-Side-NE1 \| \| CON1-Side-O1 \| \| CON1-Side-O2 \| \| TRP465-Side-NE1 \| \| CON1-Side-O8 \| \| CON1-Side-O8 \| \| TYR337-Side-OH \| | \| GLU408-Side-OE1 \| \| --- \| \| GLU408-Side-OE1 \| \| CON1-Side-O2 \| \| CON1-Side-O3 \| \| GLU194-Side-OE1 \| \| GLU464-Side-OE2 \| \| GLU408-Side-OE2 \| \| GLU194-Side-OE2 \| \| THR380-Side-OG1 \| \| PRO379-Main-O \| \| CON1-Side-O2 \| \| CON1-Side-O7 \| \| CON1-Side-O8 \| \| TRP465-Side-NE1 \| \| GLU464-Side-OE1 \| \| CON1-Side-O5 \| \| CON1-Side-O4 \| \| CON1-Side-O2 \| \| CON1-Side-O6 \| \| TRP457-Side-NE1 \| \| MET378-Side-SD \| \| GLU408-Side-OE1 \| \| CON1-Side-O3 \| \| GLU408-Side-OE2 \| \| CON1-Side-O3 \| \| TRP457-Side-NE1 \| \| GLU464-Side-OE1 \| \| MET378-Side-SD \| \| CON1-Side-O7 \| \| TYR149-Side-OH \| \| TYR337-Side-OH \| \| CON1-Side-O1 \| \| GLU355-Side-OE2 \| \| GLU355-Side-OE1 \| \| GLU194-Side-OE1 \| \| GLU194-Side-OE2 \| \| CON1-Side-O2 \| \| TYR337-Side-OH \| \| CON1-Side-O1 \| \| CON1-Side-O4 \| \| TYR149-Side-OH \| \| TYR208-Side-OH \| \| CON1-Side-O1 \| \| TYR269-Side-OH \| \| THR267-Side-OG1 \| \| CON1-Side-O4 \| | \| 64.15% \| \| --- \| \| 44.88% \| \| 57.16% \| \| 5.49% \| \| 11.50% \| \| 26.01% \| \| 0.65% \| \| 16.32% \| \| 6.83% \| \| 2.54% \| \| 0.13% \| \| 0.03% \| \| 0.43% \| \| 0.02% \| \| 20.23% \| \| 0.01% \| \| 2.67% \| \| 0.01% \| \| 0.06% \| \| 0.82% \| \| 0.06% \| \| 1.53% \| \| 0.14% \| \| 0.01% \| \| 0.06% \| \| 0.16% \| \| 1.68% \| \| 0.07% \| \| 9.01% \| \| 0.21% \| \| 0.17% \| \| 0.01% \| \| 6.32% \| \| 6.56% \| \| 7.29% \| \| 1.67% \| \| 0.28% \| \| 0.06% \| \| 0.01% \| \| 0.39% \| \| 0.09% \| \| 0.04% \| \| 0.01% \| \| 0.05% \| \| 0.05% \| \| 0.01% \| |
