# Supplementary material for: Structural analysis of rice Os4BGlu18 monolignol β-glucosidase
Source: PLoS One. 2021 Jan 20;16(1):e0241325. doi: 10.1371/journal.pone.0241325 (PMC7817009; doi:10.1371/journal.pone.0241325)
Supplement: S1 Report — (PDF) [file pone.0241325.s006.pdf]

Full wwPDB X-ray Structure Validation Report (i)

Sep 30, 2020 – 02:23 PM JST

PDB ID	:	7D6A
Title	:	Crystal structure of Oryza sativa Os4BGlu18 monolignol beta-glucosidase
Deposited on	:	2020-09-29
Resolution	:	1.70 Å(reported)

This is a Full wwPDB X-ray Structure Validation Report.

This report is produced by the wwPDB biocuration pipeline after annotation of the structure.

We welcome your comments at *validation@mail.wwpdb.org*
A user guide is available at
https://www.wwpdb.org/validation/2017/XrayValidationReportHelp
with specific help available everywhere you see the (i) symbol.

CONFIDENTIAL VALIDATION REPORT

The following versions of software and data (see references (i)) were used in the production of this report:

MolProbity	:	4.02b-467
Mogul	:	1.8.5 (274361), CSD as541be (2020)
Xtriage (Phenix)	:	1.13
EDS	:	2.14.6
buster-report	:	1.1.7 (2018)
Percentile statistics	:	20191225.v01 (using entries in the PDB archive December 25th 2019)
Refmac	:	5.8.0158
CCP4	:	7.0.044 (Gargrove)
Ideal geometry (proteins)	:	Engh & Huber (2001)
Ideal geometry (DNA, RNA)	:	Parkinson et al. (1996)
Validation Pipeline (wwPDB-VP)	:	2.14.6

1 Overall quality at a glance (i)

The following experimental techniques were used to determine the structure:
X-RAY DIFFRACTION

The reported resolution of this entry is 1.70 Å.

Percentile scores (ranging between 0-100) for global validation metrics of the entry are shown in the following graphic. The table shows the number of entries on which the scores are based.

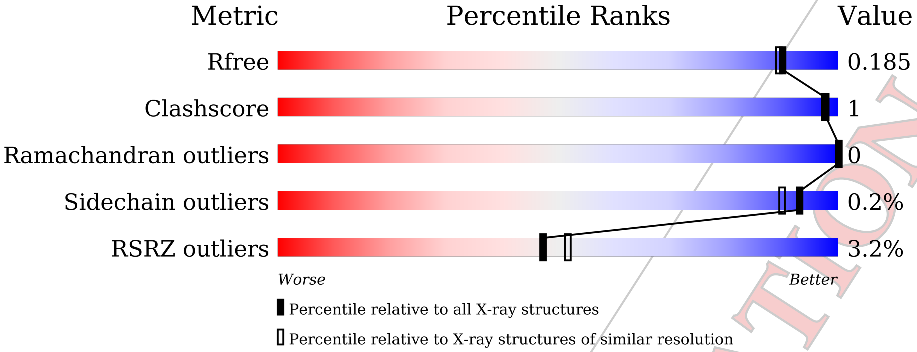

Metric	Whole archive (#Entries)	Similar resolution (#Entries, resolution range(Å))
R_{free}	130704	4298 (1.70-1.70)
Clashscore	141614	4695 (1.70-1.70)
Ramachandran outliers	138981	4610 (1.70-1.70)
Sidechain outliers	138945	4610 (1.70-1.70)
RSRZ outliers	127900	4222 (1.70-1.70)

The table below summarises the geometric issues observed across the polymeric chains and their fit to the electron density. The red, orange, yellow and green segments on the lower bar indicate the fraction of residues that contain outliers for >=3, 2, 1 and 0 types of geometric quality criteria respectively. A grey segment represents the fraction of residues that are not modelled. The numeric value for each fraction is indicated below the corresponding segment, with a dot representing fractions <=5% The upper red bar (where present) indicates the fraction of residues that have poor fit to the electron density. The numeric value is given above the bar.

Mol	Chain	Length	Quality of chain
1	A	482	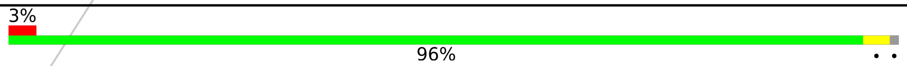
1	B	482	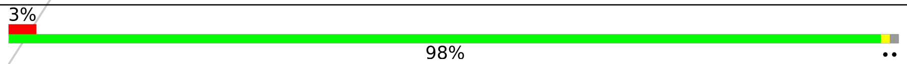

CONFIDENTIAL VALIDATION REPORT

2 Entry composition (i)

There are 5 unique types of molecules in this entry. The entry contains 8709 atoms, of which 0 are hydrogens and 0 are deuteriums.

In the tables below, the ZeroOcc column contains the number of atoms modelled with zero occupancy, the AltConf column contains the number of residues with at least one atom in alternate conformation and the Trace column contains the number of residues modelled with at most 2 atoms.

- Molecule 1 is a protein called Beta-glucosidase 18.

Mol	Chain	Residues	Atoms					ZeroOcc	AltConf	Trace
1	A	475	Total 3887	C 2496	N 655	O 721	S 15	0	5	0
1	B	477	Total 3889	C 2498	N 656	O 719	S 16	0	3	0

There are 4 discrepancies between the modelled and reference sequences:

Chain	Residue	Modelled	Actual	Comment	Reference
A	24	ALA	-	expression tag	UNP Q7XSK0
A	25	MET	-	expression tag	UNP Q7XSK0
B	24	ALA	-	expression tag	UNP Q7XSK0
B	25	MET	-	expression tag	UNP Q7XSK0

- Molecule 2 is 2-(N-MORPHOLINO)-ETHANESULFONIC ACID (three-letter code: MES) (formula: $C_6H_{13}NO_4S$) (labeled as "Ligand of Interest" by author).

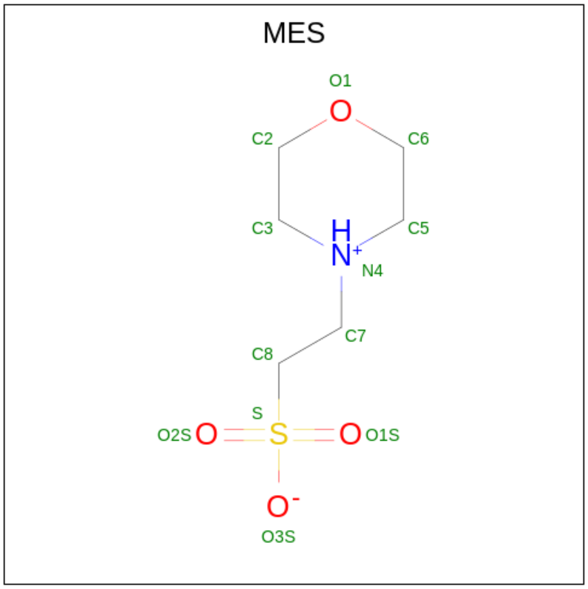

CONFIDENTIAL VALIDATION REPORT

Mol	Chain	Residues	Atoms					ZeroOcc	AltConf
2	A	1	Total 12	C 6	N 1	O 4	S 1	0	0
2	B	1	Total 12	C 6	N 1	O 4	S 1	0	0

- Molecule 3 is GLYCEROL (three-letter code: GOL) (formula: $C_3H_8O_3$) (labeled as "Ligand of Interest" by author).

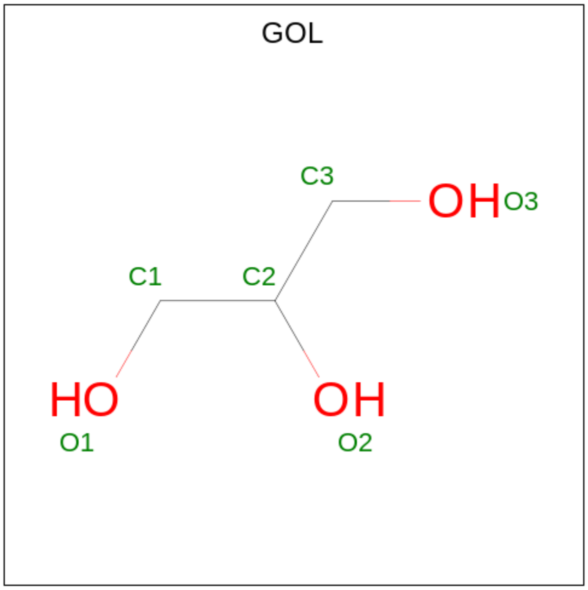

Mol	Chain	Residues	Atoms			ZeroOcc	AltConf
3	A	1	Total 6	C 3	O 3	0	0
3	A	1	Total 6	C 3	O 3	0	0
3	A	1	Total 6	C 3	O 3	0	0
3	A	1	Total 6	C 3	O 3	0	0
3	A	1	Total 6	C 3	O 3	0	0
3	A	1	Total 6	C 3	O 3	0	0
3	A	1	Total 6	C 3	O 3	0	0
3	A	1	Total 6	C 3	O 3	0	0
3	B	1	Total 6	C 3	O 3	0	0

Continued on next page...

CONFIDENTIAL VALIDATION REPORT

Continued from previous page...

Mol	Chain	Residues	Atoms	ZeroOcc	AltConf
3	B	1	Total C O<br>6 3 3	0	0
3	B	1	Total C O<br>6 3 3	0	0
3	B	1	Total C O<br>6 3 3	0	0
3	B	1	Total C O<br>6 3 3	0	0

- Molecule 4 is ZINC ION (three-letter code: ZN) (formula: Zn) (labeled as "Ligand of Interest" by author).

Mol	Chain	Residues	Atoms	ZeroOcc	AltConf
4	B	1	Total Zn<br>1 1	0	0

- Molecule 5 is water.

Mol	Chain	Residues	Atoms	ZeroOcc	AltConf
5	A	384	Total O<br>384 384	0	0
5	B	446	Total O<br>446 446	0	0

CONFIDENTIAL VALIDATION REPORT

3 Residue-property plots (i)

These plots are drawn for all protein, RNA, DNA and oligosaccharide chains in the entry. The first graphic for a chain summarises the proportions of the various outlier classes displayed in the second graphic. The second graphic shows the sequence view annotated by issues in geometry and electron density. Residues are color-coded according to the number of geometric quality criteria for which they contain at least one outlier: green = 0, yellow = 1, orange = 2 and red = 3 or more. A red dot above a residue indicates a poor fit to the electron density (RSRZ > 2). Stretches of 2 or more consecutive residues without any outlier are shown as a green connector. Residues present in the sample, but not in the model, are shown in grey.

- Molecule 1: Beta-glucosidase 18

Chain A:

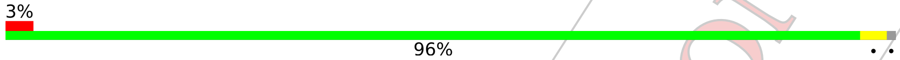

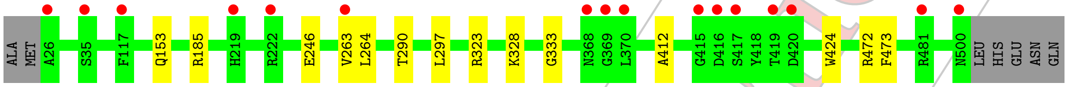

- Molecule 1: Beta-glucosidase 18

Chain B:

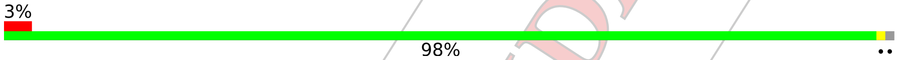

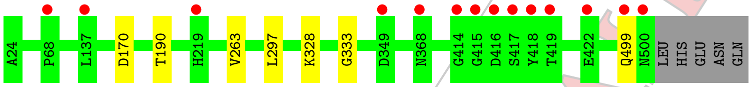

CONFIDENTIAL VALIDATION REPORT

4 Data and refinement statistics (i)

Property	Value	Source
Space group	P 21 21 21	Depositor
Cell constants<br>a, b, c, α, β, γ	52.13Å 83.76Å 207.65Å<br>90.00° 90.00° 90.00°	Depositor
Resolution (Å)	30.00 – 1.70<br>27.96 – 1.70	Depositor<br>EDS
% Data completeness<br>(in resolution range)	95.9 (30.00-1.70)<br>95.9 (27.96-1.70)	Depositor<br>EDS
R_{merge}	(Not available)	Depositor
R_{sym}	(Not available)	Depositor
$< I/\sigma(I) >$ [1]	2.46 (at 1.70Å)	Xtriage
Refinement program	REFMAC 5.8.0135	Depositor
R, R_{free}	0.150 , 0.175<br>0.164 , 0.185	Depositor<br>DCC
R_{free} test set	4863 reflections (5.03%)	wwPDB-VP
Wilson B-factor (Å^2)	17.2	Xtriage
Anisotropy	0.026	Xtriage
Bulk solvent k_{sol}(e/Å^3), B_{sol}(Å^2)	0.38 , 42.5	EDS
L-test for twinning[2]	$< \|L\| > = 0.49$, $< L^2 > = 0.32$	Xtriage
Estimated twinning fraction	No twinning to report.	Xtriage
F_o,F_c correlation	0.97	EDS
Total number of atoms	8709	wwPDB-VP
Average B, all atoms (Å^2)	19.0	wwPDB-VP

Xtriage's analysis on translational NCS is as follows: *The largest off-origin peak in the Patterson function is 3.87% of the height of the origin peak. No significant pseudotranslation is detected.*

[1] Intensities estimated from amplitudes.

[2] Theoretical values of $< |L| >$, $< L^2 >$ for acentric reflections are 0.5, 0.333 respectively for untwinned datasets, and 0.375, 0.2 for perfectly twinned datasets.

CONFIDENTIAL VALIDATION REPORT

5 Model quality (i)

5.1 Standard geometry (i)

Bond lengths and bond angles in the following residue types are not validated in this section: GOL, ZN, MES

The Z score for a bond length (or angle) is the number of standard deviations the observed value is removed from the expected value. A bond length (or angle) with $|Z| > 5$ is considered an outlier worth inspection. RMSZ is the root-mean-square of all Z scores of the bond lengths (or angles).

Mol	Chain	Bond lengths		Bond angles	
		RMSZ	#$\|Z\|$ >5	RMSZ	#$\|Z\|$ >5
1	A	0.41	0/4016	0.67	1/5457 (0.0%)
1	B	0.41	0/4015	0.66	0/5455
All	All	0.41	0/8031	0.67	1/10912 (0.0%)

There are no bond length outliers.

All (1) bond angle outliers are listed below:

Mol	Chain	Res	Type	Atoms	Z	Observed(^{o})	Ideal(^{o})
1	A	185	ARG	NE-CZ-NH1	5.02	122.81	120.30

There are no chirality outliers.

There are no planarity outliers.

5.2 Too-close contacts (i)

In the following table, the Non-H and H(model) columns list the number of non-hydrogen atoms and hydrogen atoms in the chain respectively. The H(added) column lists the number of hydrogen atoms added and optimized by MolProbity. The Clashes column lists the number of clashes within the asymmetric unit, whereas Symm-Clashes lists symmetry related clashes.

Mol	Chain	Non-H	H(model)	H(added)	Clashes	Symm-Clashes
1	A	3887	0	3677	7	0
1	B	3889	0	3686	3	0
2	A	12	0	13	0	0
2	B	12	0	13	0	0
3	A	48	0	64	1	0
3	B	30	0	40	0	0
4	B	1	0	0	0	0
5	A	384	0	0	0	0

Continued on next page...

CONFIDENTIAL VALIDATION REPORT

Continued from previous page...

Mol	Chain	Non-H	H(model)	H(added)	Clashes	Symm-Clashes
5	B	446	0	0	0	0
All	All	8709	0	7493	10	0

The all-atom clashscore is defined as the number of clashes found per 1000 atoms (including hydrogen atoms). The all-atom clashscore for this structure is 1.

All (10) close contacts within the same asymmetric unit are listed below, sorted by their clash magnitude.

Atom-1	Atom-2	Interatomic distance (Å)	Clash overlap (Å)
1:A:153:GLN:OE1	3:A:604:GOL:H11	2.06	0.55
1:A:264:LEU:HB3	1:A:290:THR:HG22	1.96	0.48
1:A:263:VAL:HG22	1:A:333:GLY:HA3	1.96	0.47
1:B:190:THR:HG21	1:B:263:VAL:HG23	1.96	0.46
1:A:246:GLU:OE2	1:A:323:ARG:NH1	2.51	0.43
1:A:297:LEU:O	1:A:328:LYS:HA	2.20	0.42
1:B:263:VAL:HG22	1:B:333:GLY:HA3	2.02	0.42
1:A:412:ALA:HB2	1:A:473:PHE:CZ	2.55	0.42
1:B:297:LEU:O	1:B:328:LYS:HA	2.21	0.40
1:A:424:TRP:CE3	1:A:472:ARG:HD2	2.57	0.40

There are no symmetry-related clashes.

5.3 Torsion angles (i)

5.3.1 Protein backbone (i)

In the following table, the Percentiles column shows the percent Ramachandran outliers of the chain as a percentile score with respect to all X-ray entries followed by that with respect to entries of similar resolution.

The Analysed column shows the number of residues for which the backbone conformation was analysed, and the total number of residues.

Mol	Chain	Analysed	Favoured	Allowed	Outliers	Percentiles	
1	A	478/482 (99%)	466 (98%)	12 (2%)	0	100	100
1	B	478/482 (99%)	466 (98%)	12 (2%)	0	100	100
All	All	956/964 (99%)	932 (98%)	24 (2%)	0	100	100

There are no Ramachandran outliers to report.

CONFIDENTIAL VALIDATION REPORT

5.3.2 Protein sidechains (i)

In the following table, the Percentiles column shows the percent sidechain outliers of the chain as a percentile score with respect to all X-ray entries followed by that with respect to entries of similar resolution.

The Analysed column shows the number of residues for which the sidechain conformation was analysed, and the total number of residues.

Mol	Chain	Analysed	Rotameric	Outliers	Percentiles	
1	A	407/409 (100%)	407 (100%)	0	100	100
1	B	406/409 (99%)	404 (100%)	2 (0%)	88	83
All	All	813/818 (99%)	811 (100%)	2 (0%)	93	90

All (2) residues with a non-rotameric sidechain are listed below:

Mol	Chain	Res	Type
1	B	170	ASP
1	B	499	GLN

Some sidechains can be flipped to improve hydrogen bonding and reduce clashes. There are no such sidechains identified.

5.3.3 RNA (i)

There are no RNA molecules in this entry.

5.4 Non-standard residues in protein, DNA, RNA chains (i)

There are no non-standard protein/DNA/RNA residues in this entry.

5.5 Carbohydrates (i)

There are no monosaccharides in this entry.

5.6 Ligand geometry (i)

Of 16 ligands modelled in this entry, 1 is monoatomic - leaving 15 for Mogul analysis.

In the following table, the Counts columns list the number of bonds (or angles) for which Mogul statistics could be retrieved, the number of bonds (or angles) that are observed in the model and the number of bonds (or angles) that are defined in the Chemical Component Dictionary. The

CONFIDENTIAL VALIDATION REPORT

Link column lists molecule types, if any, to which the group is linked. The Z score for a bond length (or angle) is the number of standard deviations the observed value is removed from the expected value. A bond length (or angle) with $|Z| > 2$ is considered an outlier worth inspection. RMSZ is the root-mean-square of all Z scores of the bond lengths (or angles).

Mol	Type	Chain	Res	Link	Bond lengths			Bond angles		
					Counts	RMSZ	$\#\|Z\| > 2$	Counts	RMSZ	$\#\|Z\| > 2$
3	GOL	B	604	-	5,5,5	0.40	0	5,5,5	0.35	0
3	GOL	A	609	-	5,5,5	0.20	0	5,5,5	0.52	0
3	GOL	A	606	-	5,5,5	0.32	0	5,5,5	0.37	0
3	GOL	A	605	-	5,5,5	0.44	0	5,5,5	0.57	0
3	GOL	A	602	-	5,5,5	0.41	0	5,5,5	0.34	0
3	GOL	B	605	-	5,5,5	0.34	0	5,5,5	0.46	0
3	GOL	A	608	-	5,5,5	0.30	0	5,5,5	0.42	0
3	GOL	B	606	-	5,5,5	0.27	0	5,5,5	0.23	0
3	GOL	A	604	-	5,5,5	0.34	0	5,5,5	0.73	0
3	GOL	B	607	-	5,5,5	0.32	0	5,5,5	0.23	0
3	GOL	A	607	-	5,5,5	0.18	0	5,5,5	0.47	0
2	MES	B	602	-	12,12,12	2.23	1 (8%)	14,16,16	1.17	2 (14%)
3	GOL	A	603	-	5,5,5	0.26	0	5,5,5	0.41	0
3	GOL	B	603	-	5,5,5	0.18	0	5,5,5	0.43	0
2	MES	A	601	-	12,12,12	2.19	1 (8%)	14,16,16	6.11	9 (64%)

In the following table, the Chirals column lists the number of chiral outliers, the number of chiral centers analysed, the number of these observed in the model and the number defined in the Chemical Component Dictionary. Similar counts are reported in the Torsion and Rings columns. '-' means no outliers of that kind were identified.

Mol	Type	Chain	Res	Link	Chirals	Torsions	Rings
3	GOL	B	604	-	-	0/4/4/4	-
3	GOL	A	609	-	-	2/4/4/4	-
3	GOL	A	606	-	-	0/4/4/4	-
3	GOL	A	605	-	-	2/4/4/4	-
3	GOL	A	602	-	-	0/4/4/4	-
3	GOL	B	605	-	-	0/4/4/4	-
3	GOL	A	608	-	-	0/4/4/4	-
3	GOL	B	606	-	-	0/4/4/4	-
3	GOL	A	604	-	-	2/4/4/4	-
3	GOL	B	607	-	-	2/4/4/4	-
3	GOL	A	607	-	-	2/4/4/4	-
2	MES	B	602	-	-	1/6/14/14	0/1/1/1
3	GOL	A	603	-	-	2/4/4/4	-
3	GOL	B	603	-	-	0/4/4/4	-

Continued on next page...

CONFIDENTIAL VALIDATION REPORT

Continued from previous page...

Mol	Type	Chain	Res	Link	Chirals	Torsions	Rings
2	MES	A	601	-	-	3/6/14/14	0/1/1/1

All (2) bond length outliers are listed below:

Mol	Chain	Res	Type	Atoms	Z	Observed(Å)	Ideal(Å)
2	B	602	MES	C8-S	-7.40	1.67	1.77
2	A	601	MES	C8-S	-7.09	1.67	1.77

All (11) bond angle outliers are listed below:

Mol	Chain	Res	Type	Atoms	Z	Observed(^{o})	Ideal(^{o})
2	A	601	MES	O3S-S-O2S	-11.98	82.00	111.27
2	A	601	MES	O2S-S-C8	11.63	120.92	106.92
2	A	601	MES	O3S-S-O1S	-9.75	87.44	111.27
2	A	601	MES	O1S-S-C8	7.77	116.28	106.92
2	A	601	MES	O3S-S-C8	-7.56	93.55	105.77
2	A	601	MES	C5-N4-C3	2.84	115.22	108.83
2	B	602	MES	O3S-S-C8	2.80	110.30	105.77
2	A	601	MES	C6-C5-N4	2.76	114.29	110.10
2	A	601	MES	O2S-S-O1S	2.40	122.25	113.95
2	A	601	MES	C2-C3-N4	2.36	113.67	110.10
2	B	602	MES	O1S-S-C8	2.27	109.65	106.92

There are no chirality outliers.

All (16) torsion outliers are listed below:

Mol	Chain	Res	Type	Atoms
3	A	605	GOL	C1-C2-C3-O3
3	A	605	GOL	O2-C2-C3-O3
3	A	604	GOL	O1-C1-C2-O2
3	A	604	GOL	O1-C1-C2-C3
2	A	601	MES	C8-C7-N4-C5
2	A	601	MES	C7-C8-S-O2S
3	A	607	GOL	O1-C1-C2-C3
2	B	602	MES	N4-C7-C8-S
3	A	603	GOL	C1-C2-C3-O3
3	A	603	GOL	O2-C2-C3-O3
3	A	609	GOL	C1-C2-C3-O3
3	B	607	GOL	O1-C1-C2-C3
3	A	609	GOL	O2-C2-C3-O3
3	A	607	GOL	O1-C1-C2-O2

Continued on next page...

CONFIDENTIAL VALIDATION REPORT

Continued from previous page...

Mol	Chain	Res	Type	Atoms
2	A	601	MES	C7-C8-S-O1S
3	B	607	GOL	O1-C1-C2-O2

There are no ring outliers.

1 monomer is involved in 1 short contact:

Mol	Chain	Res	Type	Clashes	Symm-Clashes
3	A	604	GOL	1	0

The following is a two-dimensional graphical depiction of Mogul quality analysis of bond lengths, bond angles, torsion angles, and ring geometry for all instances of the Ligand of Interest. In addition, ligands with molecular weight > 250 and outliers as shown on the validation Tables will also be included. For torsion angles, if less then 5% of the Mogul distribution of torsion angles is within 10 degrees of the torsion angle in question, then that torsion angle is considered an outlier. Any bond that is central to one or more torsion angles identified as an outlier by Mogul will be highlighted in the graph. For rings, the root-mean-square deviation (RMSD) between the ring in question and similar rings identified by Mogul is calculated over all ring torsion angles. If the average RMSD is greater than 60 degrees and the minimal RMSD between the ring in question and any Mogul-identified rings is also greater than 60 degrees, then that ring is considered an outlier. The outliers are highlighted in purple. The color gray indicates Mogul did not find sufficient equivalents in the CSD to analyse the geometry.

CONFIDENTIAL VALIDATION REPORT

Ligand GOL B 604

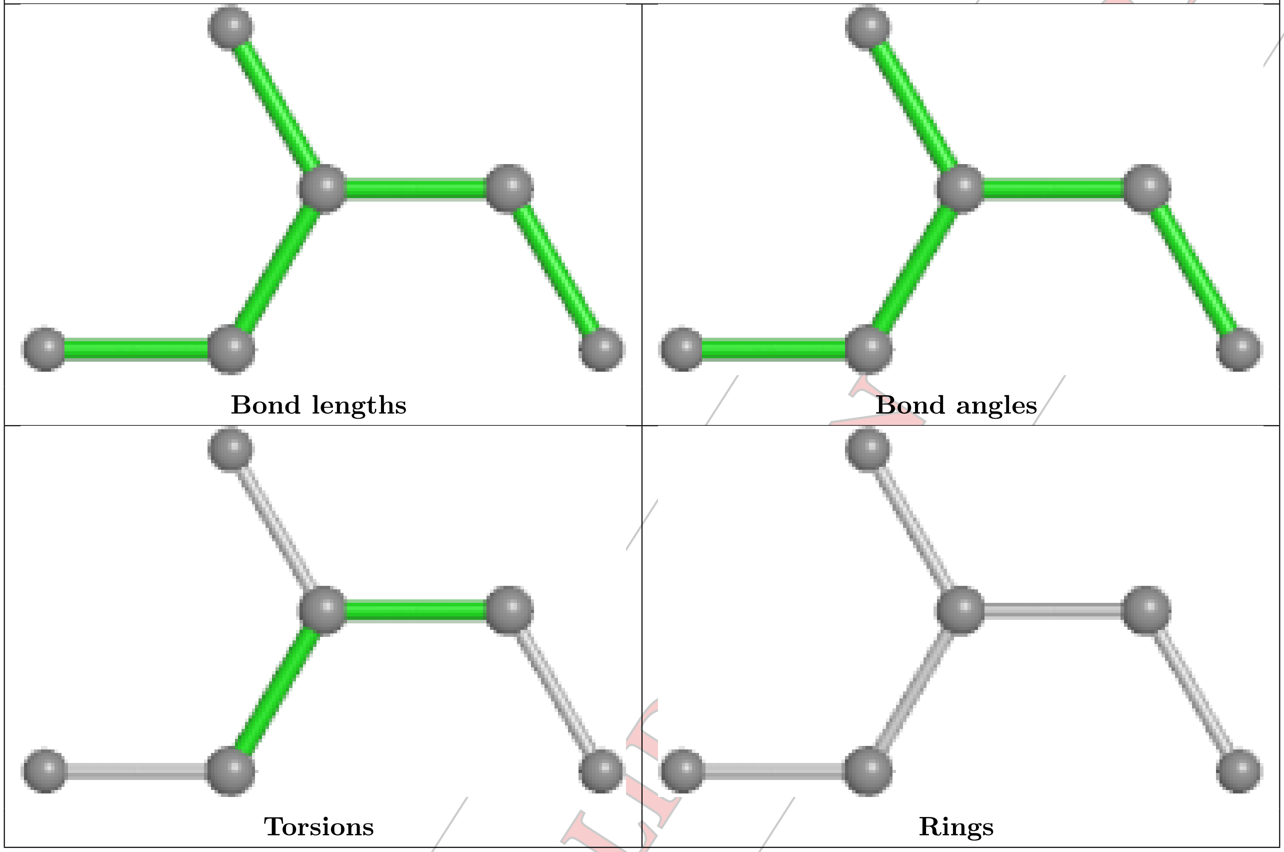

Ligand GOL A 609

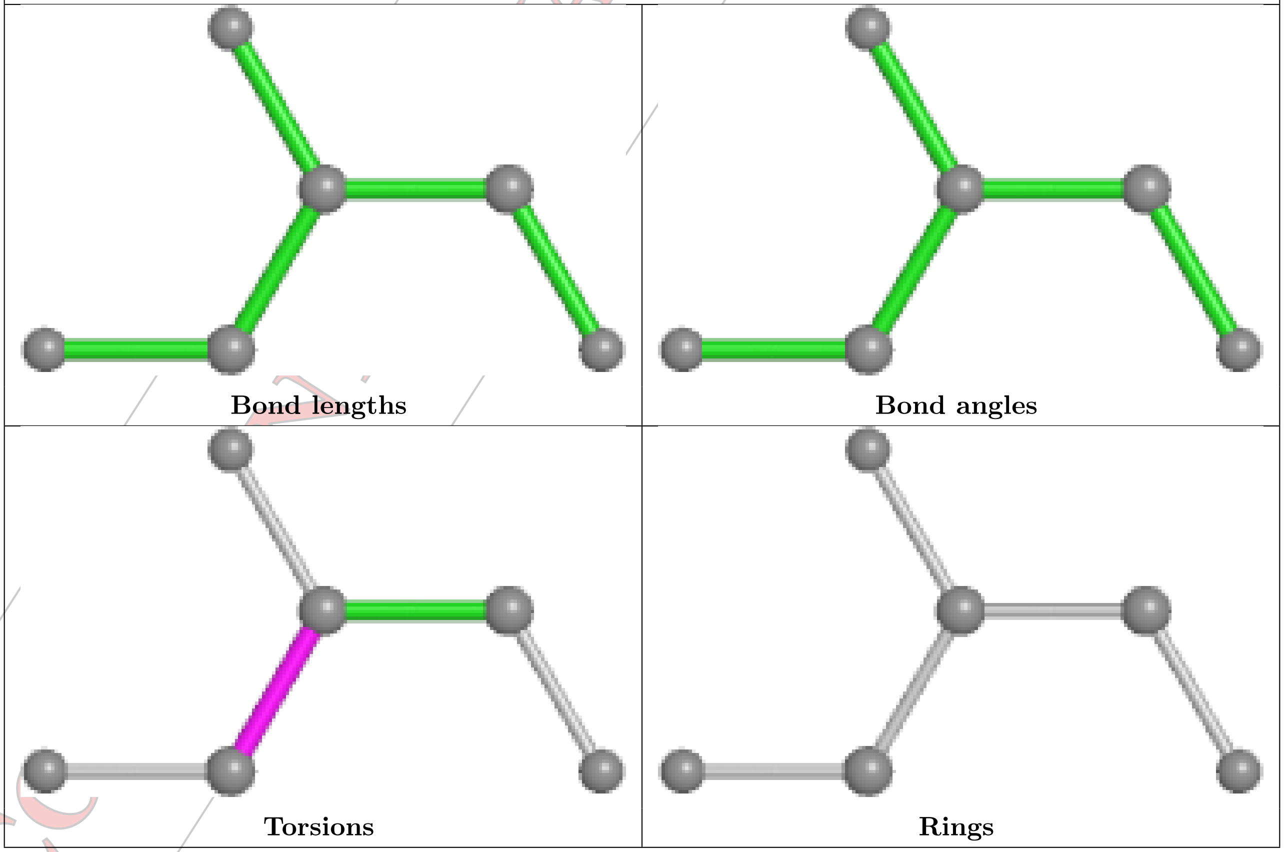

Ligand GOL A 606

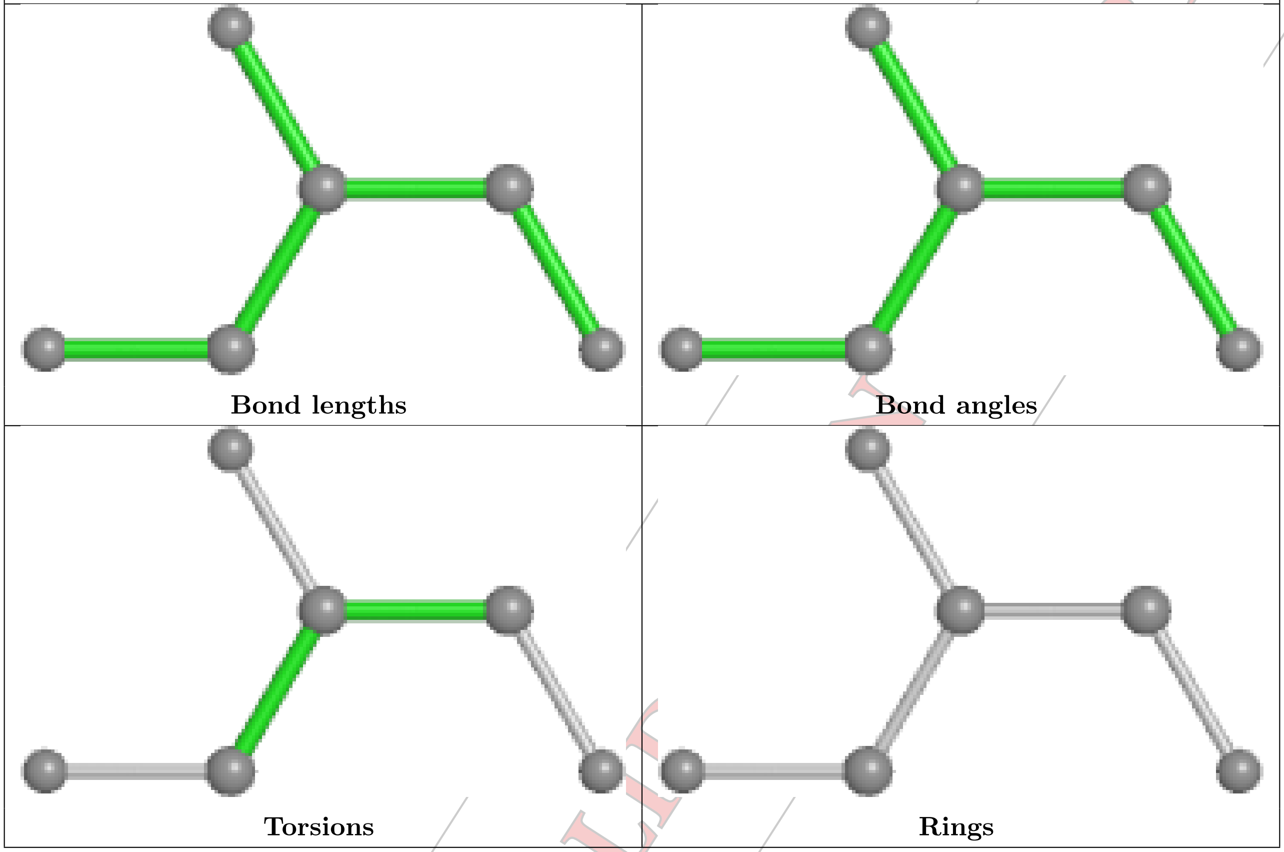

Ligand GOL A 605

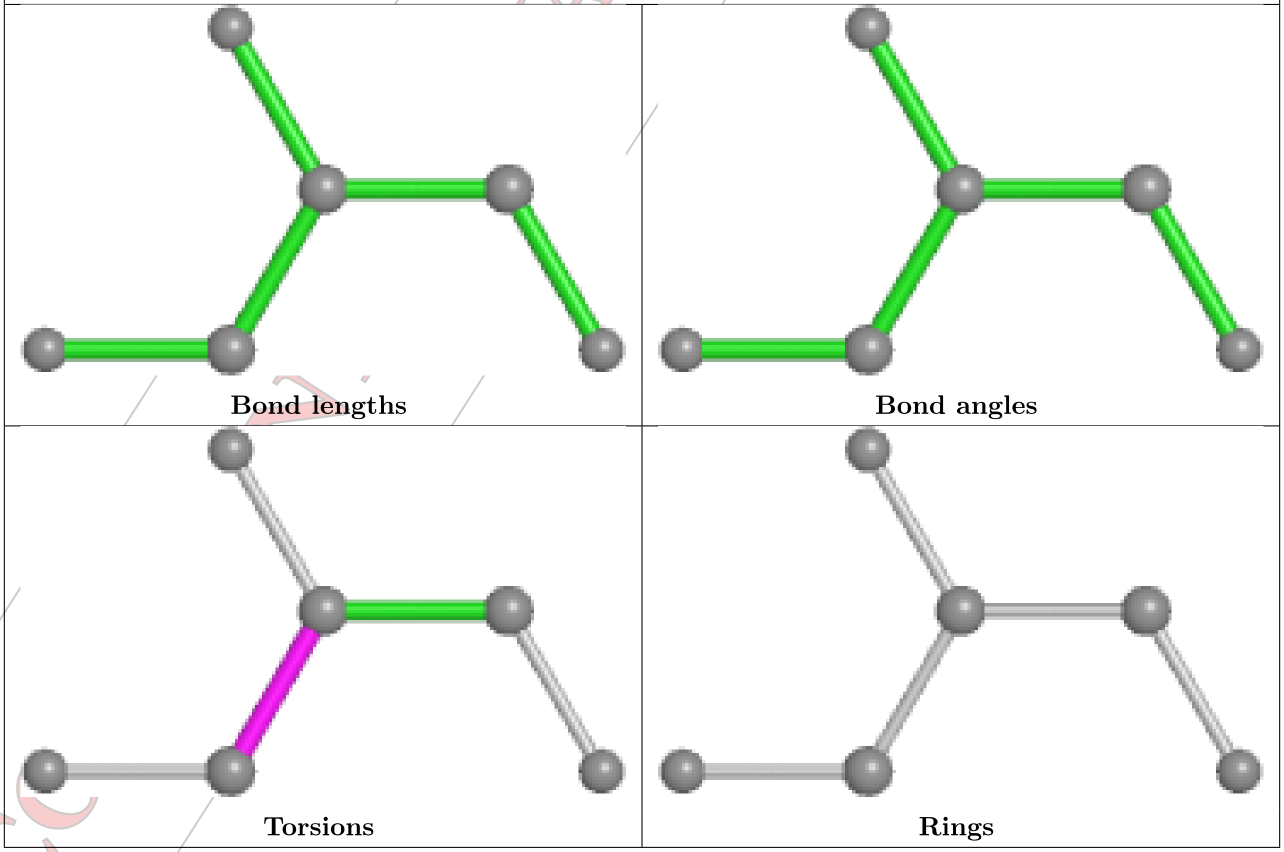

Ligand GOL A 602

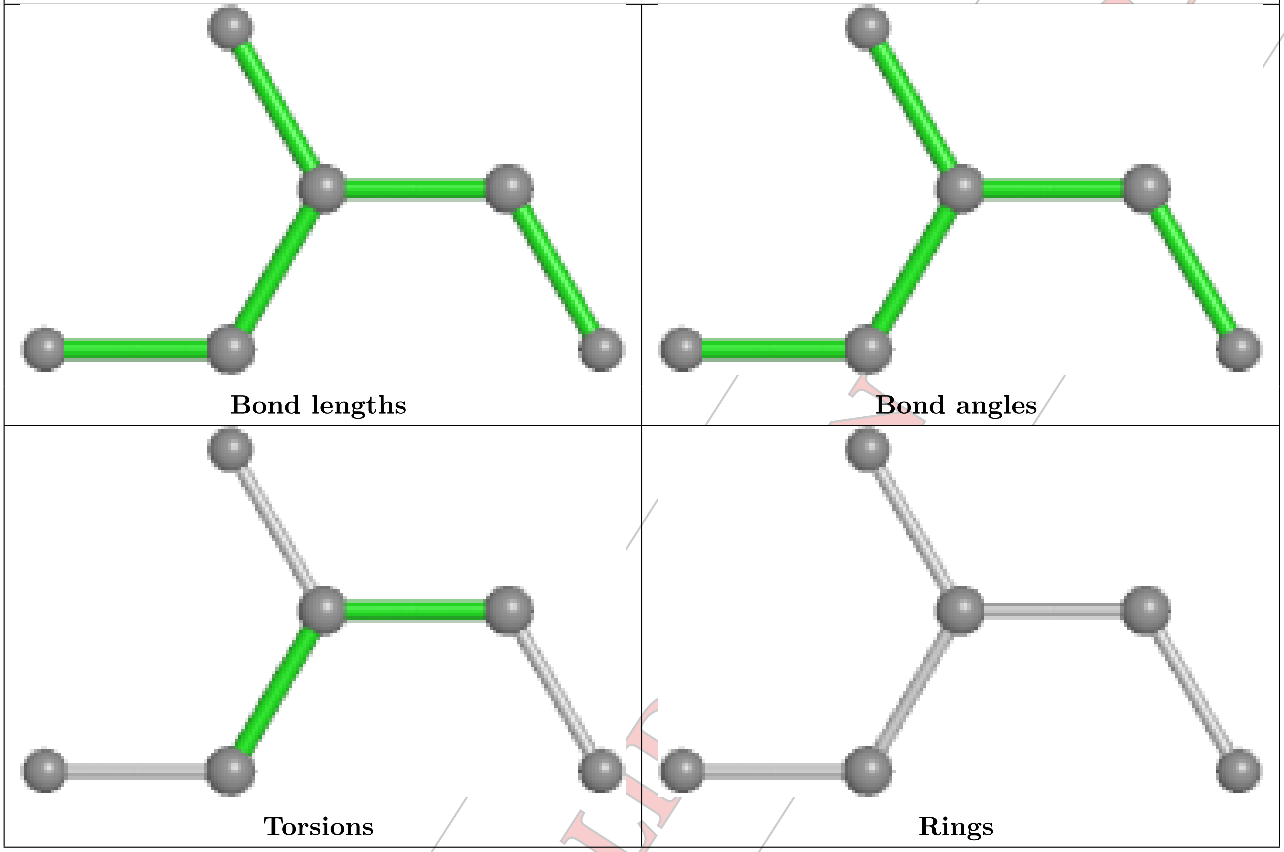

Ligand GOL B 605

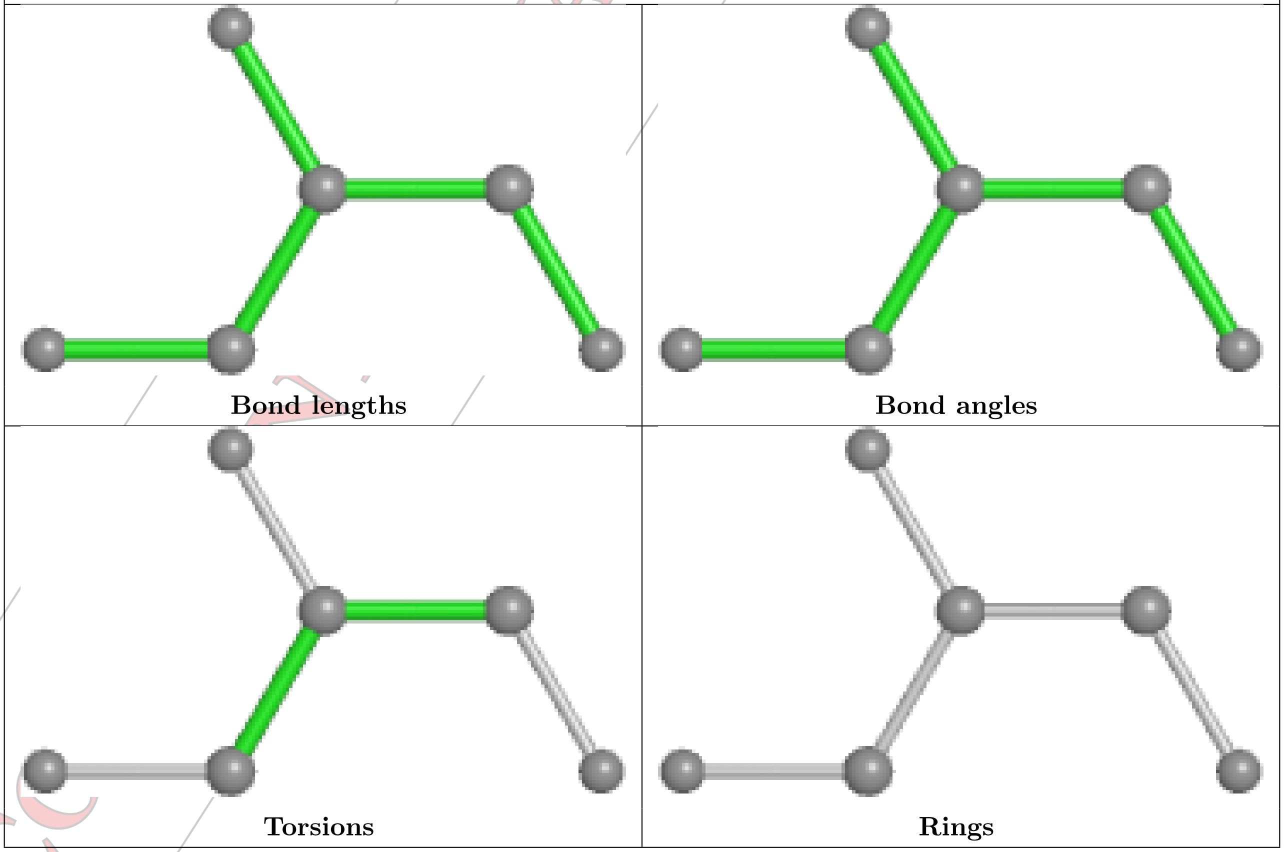

Ligand GOL A 608

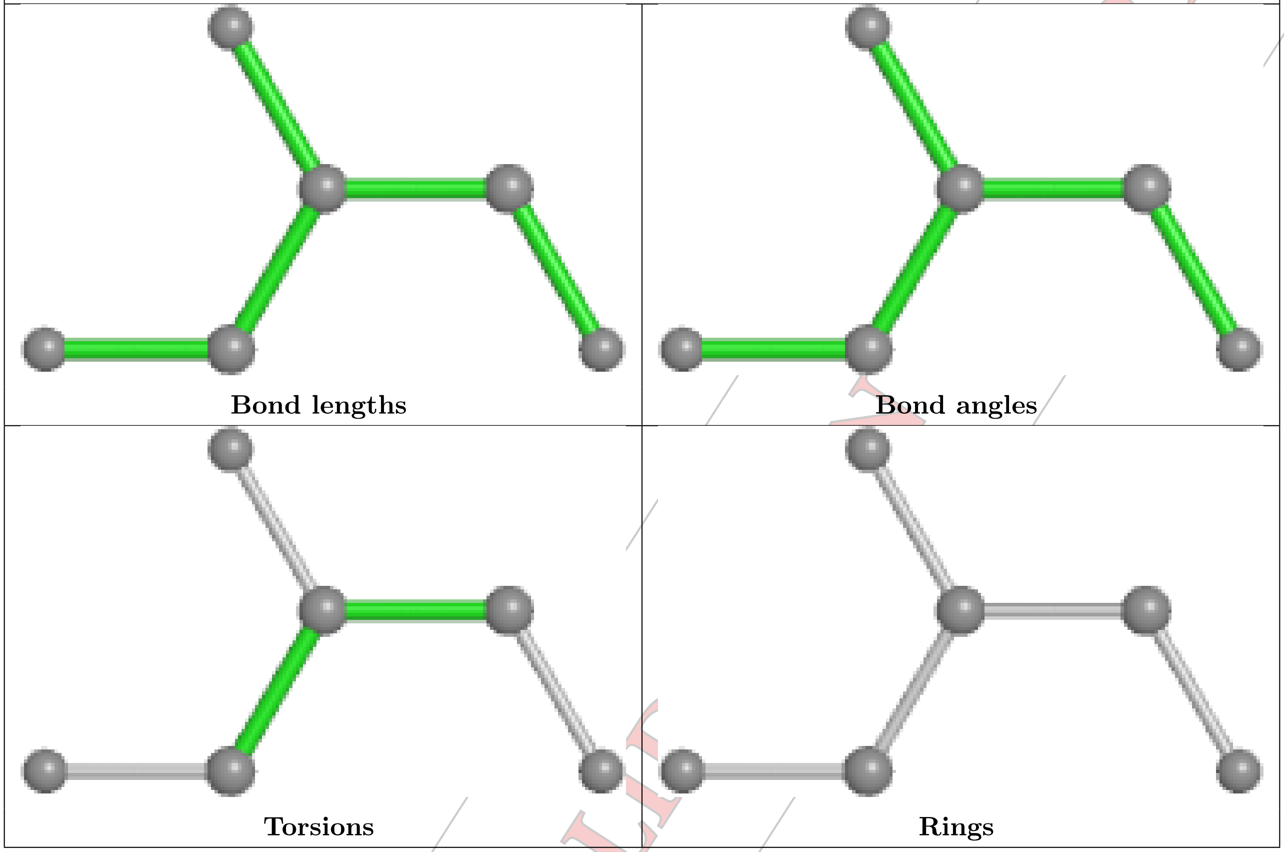

Ligand GOL B 606

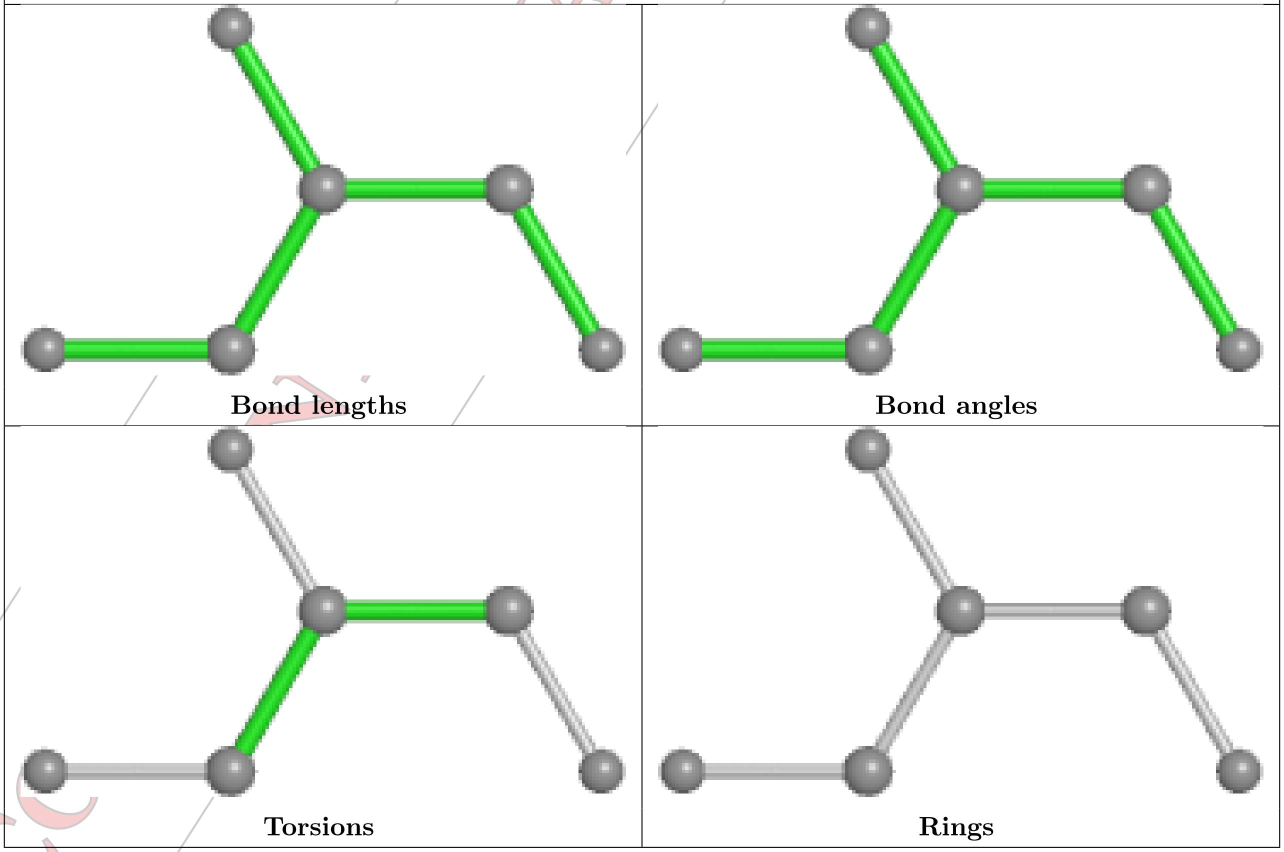

Ligand GOL A 604

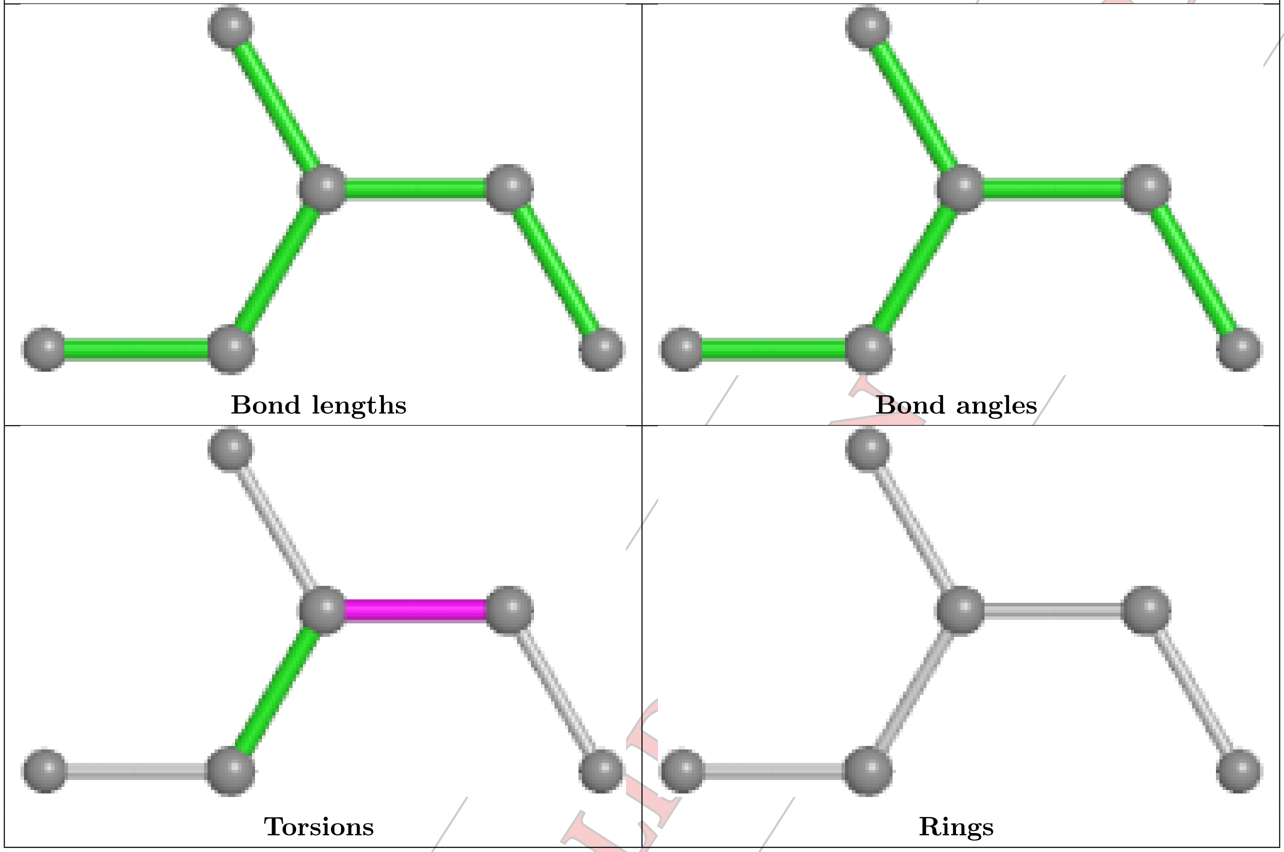

Ligand GOL B 607

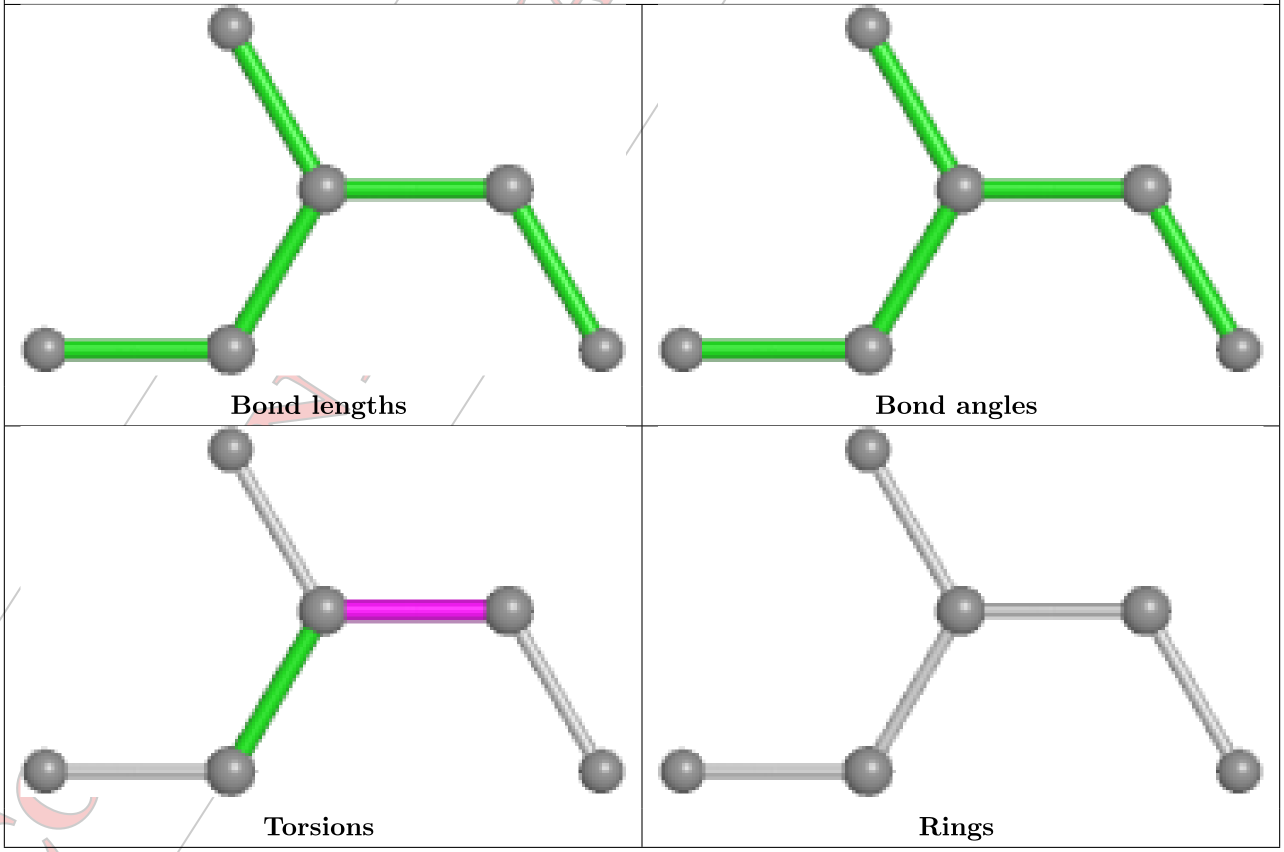

Ligand GOL A 607

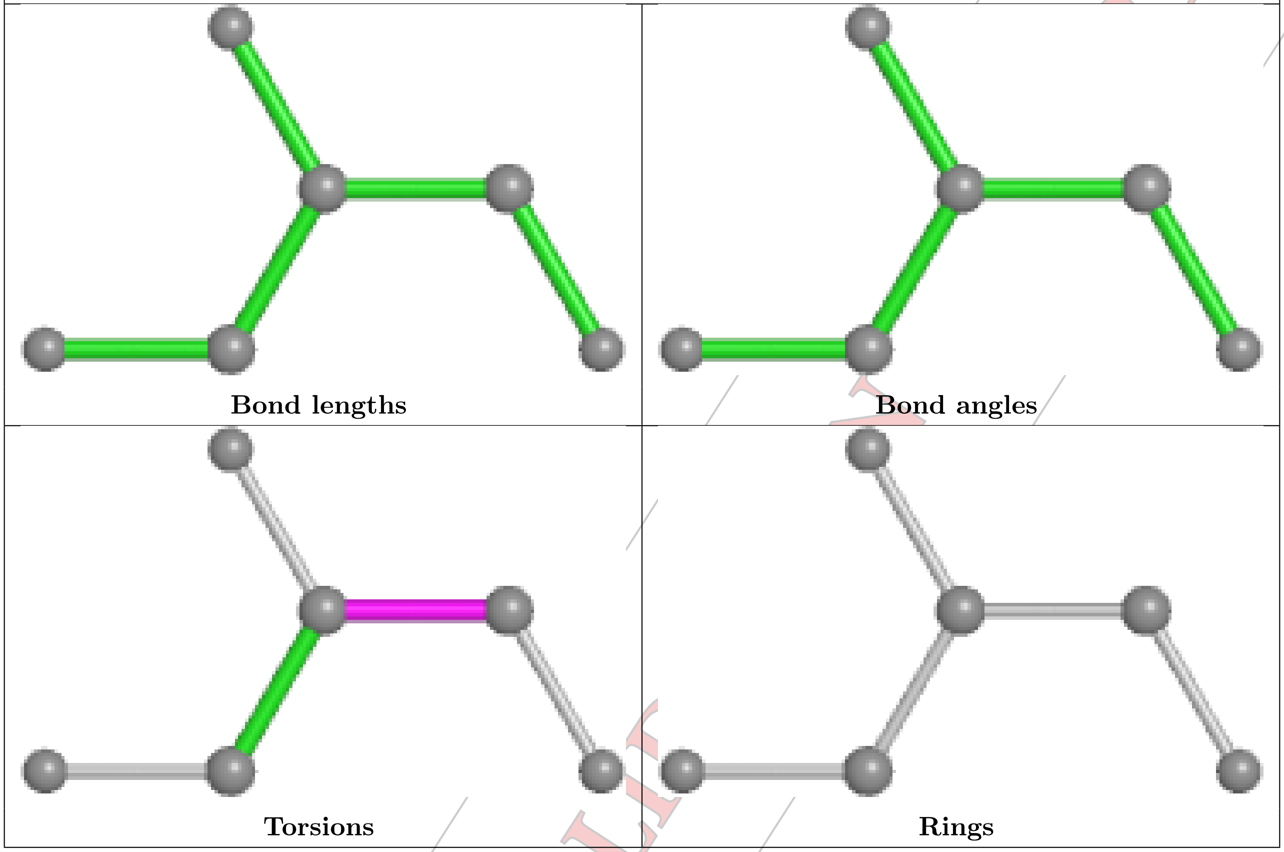

Ligand MES B 602

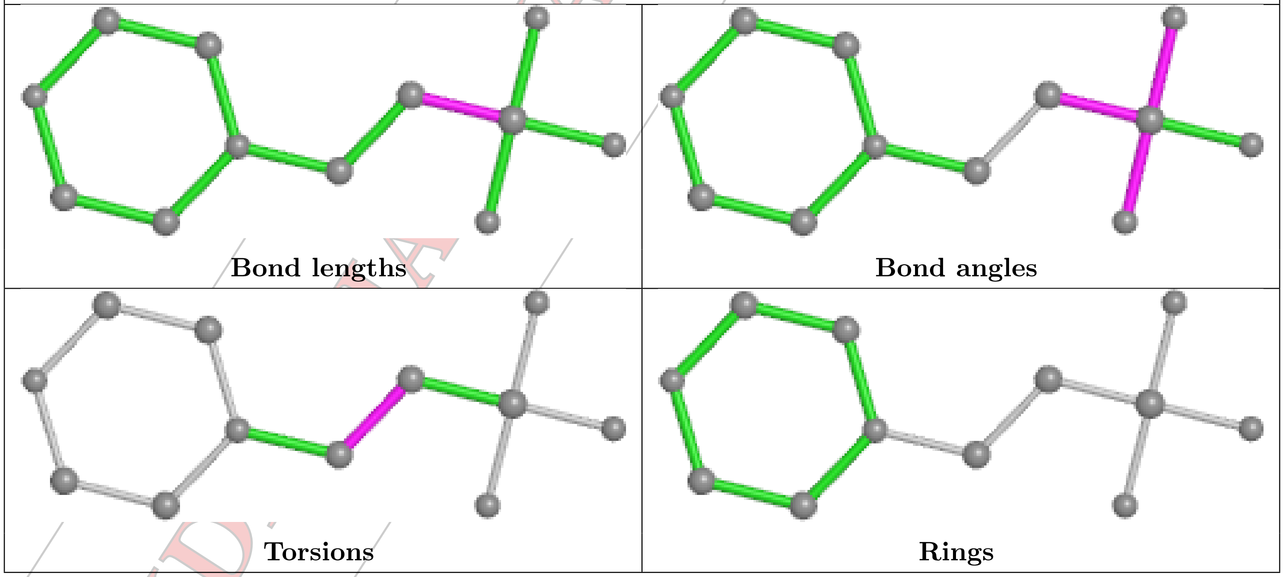

Ligand GOL A 603

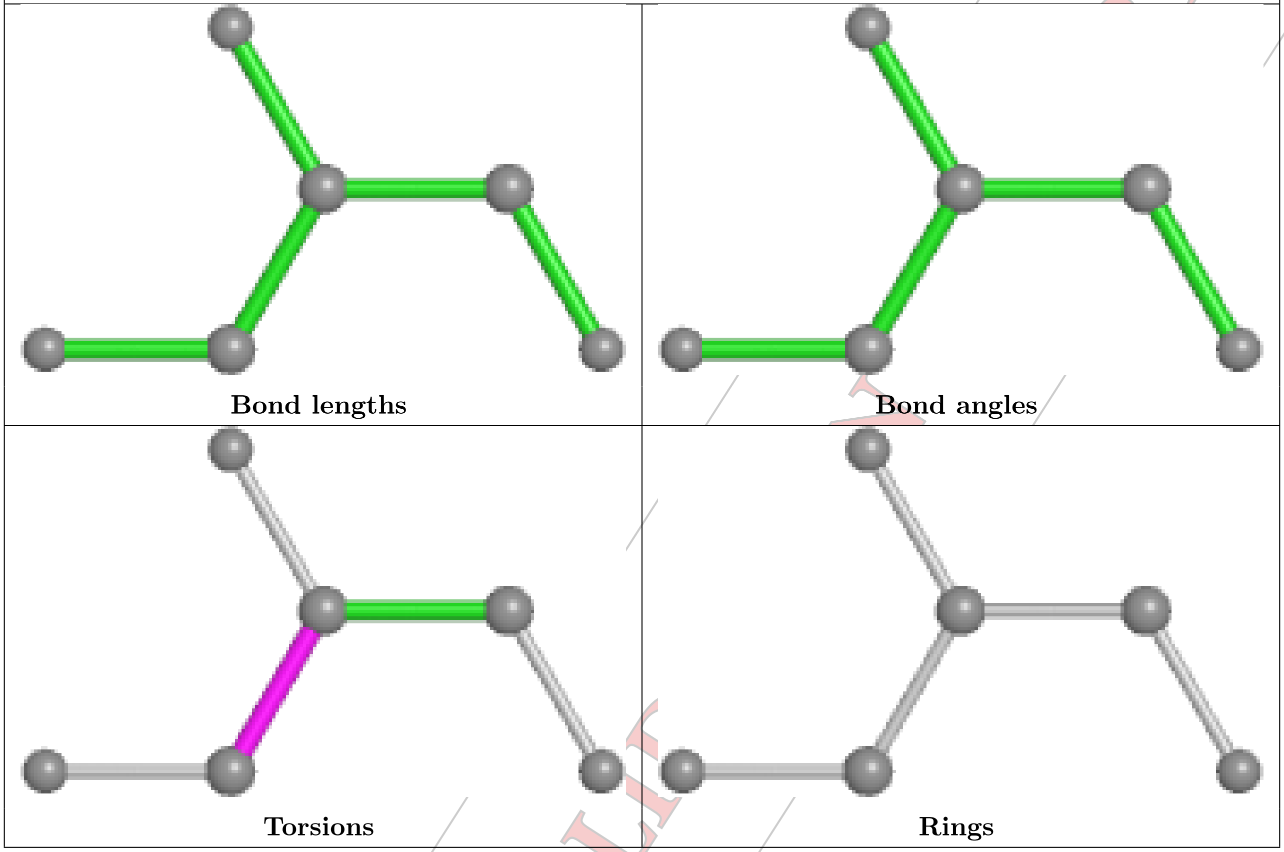

Ligand GOL B 603

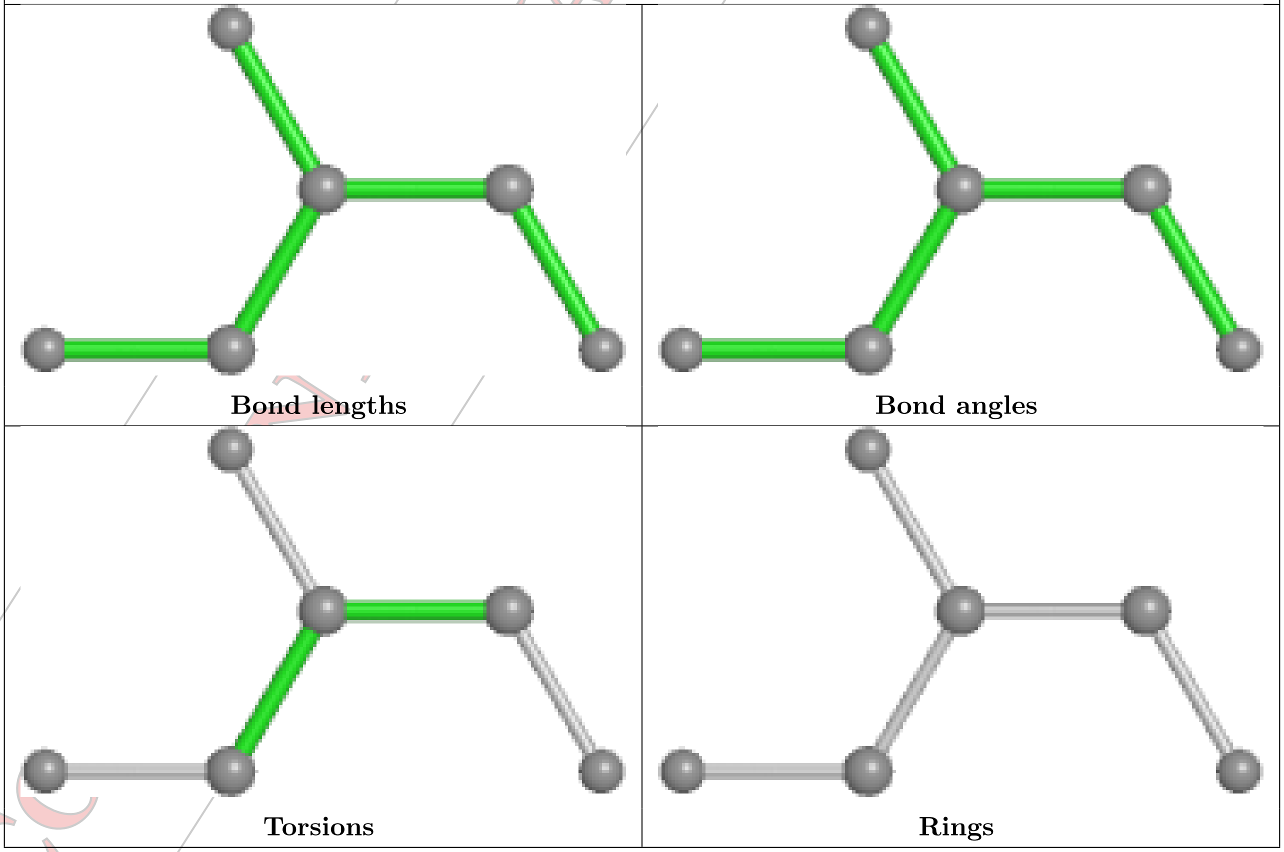

Ligand MES A 601

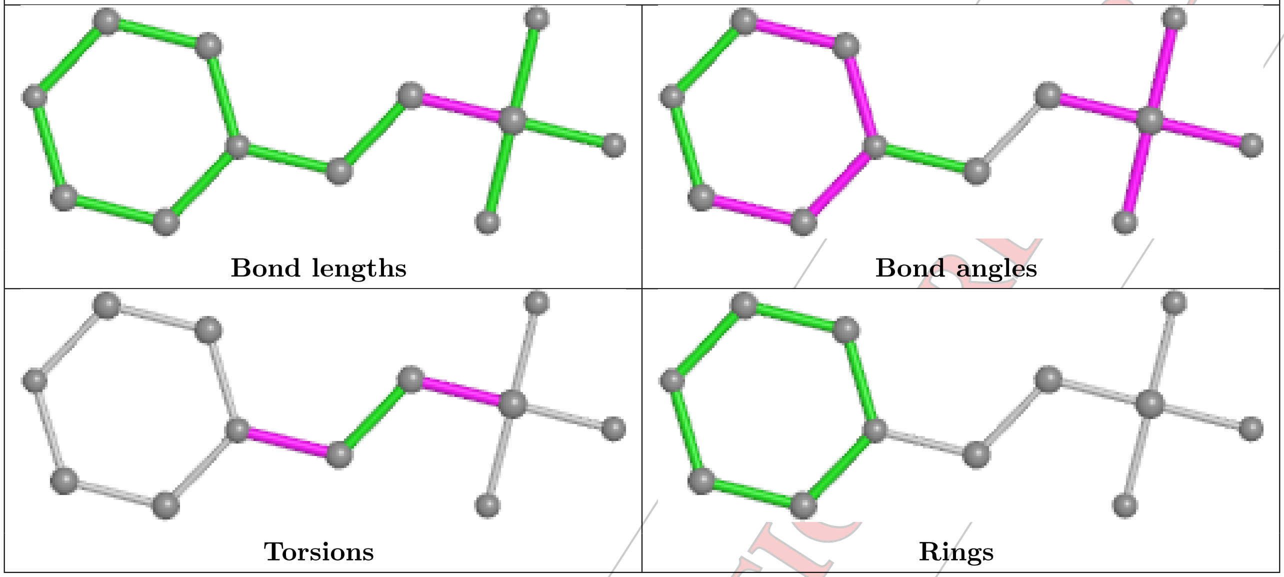

Bond lengths | Bond angles | Torsions | Rings

5.7 Other polymers (i)

There are no such residues in this entry.

5.8 Polymer linkage issues (i)

There are no chain breaks in this entry.

CONFIDENTIAL VALIDATION REPORT

6 Fit of model and data (i)

6.1 Protein, DNA and RNA chains (i)

In the following table, the column labelled '#RSRZ> 2' contains the number (and percentage) of RSRZ outliers, followed by percent RSRZ outliers for the chain as percentile scores relative to all X-ray entries and entries of similar resolution. The OWAB column contains the minimum, median, 95th percentile and maximum values of the occupancy-weighted average B-factor per residue. The column labelled 'Q< 0.9' lists the number of (and percentage) of residues with an average occupancy less than 0.9.

Mol	Chain	Analysed	<RSRZ>	#RSRZ>2			OWAB(Å^2)	Q<0.9
1	A	475/482 (98%)	-0.07	16 (3%)	45	50	11, 17, 31, 45	0
1	B	477/482 (98%)	-0.13	14 (2%)	51	56	12, 16, 27, 53	0
All	All	952/964 (98%)	-0.10	30 (3%)	47	52	11, 17, 29, 53	0

All (30) RSRZ outliers are listed below:

Mol	Chain	Res	Type	RSRZ
1	A	117	PHE	6.2
1	A	419	THR	5.9
1	A	370	LEU	5.4
1	B	418	TYR	5.1
1	A	417	SER	4.7
1	B	417	SER	4.5
1	B	500	ASN	4.4
1	B	419	THR	4.4
1	A	369	GLY	4.2
1	B	416	ASP	4.1
1	A	219	HIS	3.6
1	A	416	ASP	3.0
1	A	415	GLY	3.0
1	A	368	ASN	2.8
1	B	415	GLY	2.7
1	A	222	ARG	2.7
1	A	263	VAL	2.6
1	A	35	SER	2.6
1	B	499	GLN	2.6
1	B	414	GLY	2.5
1	B	68	PRO	2.5
1	B	137	LEU	2.5
1	A	500	ASN	2.4
1	B	219	HIS	2.4

Continued on next page...

CONFIDENTIAL VALIDATION REPORT

Continued from previous page...

Mol	Chain	Res	Type	RSRZ
1	A	481	ARG	2.4
1	A	26	ALA	2.2
1	B	349	ASP	2.2
1	B	422	GLU	2.1
1	A	420	ASP	2.1
1	B	368	ASN	2.0

6.2 Non-standard residues in protein, DNA, RNA chains (i)

There are no non-standard protein/DNA/RNA residues in this entry.

6.3 Carbohydrates (i)

There are no monosaccharides in this entry.

6.4 Ligands (i)

In the following table, the Atoms column lists the number of modelled atoms in the group and the number defined in the chemical component dictionary. The B-factors column lists the minimum, median, 95th percentile and maximum values of B factors of atoms in the group. The column labelled 'Q< 0.9' lists the number of atoms with occupancy less than 0.9.

Mol	Type	Chain	Res	Atoms	RSCC	RSR	B-factors(Å^2)	Q<0.9
3	GOL	A	605	6/6	0.70	0.29	41,47,49,50	0
3	GOL	A	607	6/6	0.70	0.22	36,39,41,43	0
3	GOL	B	607	6/6	0.79	0.22	37,41,42,44	0
3	GOL	A	604	6/6	0.79	0.28	33,36,36,40	0
3	GOL	B	606	6/6	0.83	0.16	27,32,35,36	0
3	GOL	A	603	6/6	0.83	0.18	20,27,28,33	0
2	MES	A	601	12/12	0.84	0.17	27,29,35,37	0
3	GOL	A	609	6/6	0.88	0.16	25,30,33,37	0
3	GOL	A	606	6/6	0.90	0.17	24,29,32,36	0
2	MES	B	602	12/12	0.93	0.13	23,24,28,28	0
3	GOL	B	604	6/6	0.94	0.14	16,17,18,20	0
3	GOL	A	602	6/6	0.94	0.11	15,16,17,20	0
3	GOL	A	608	6/6	0.96	0.09	16,17,18,19	0
3	GOL	B	603	6/6	0.97	0.06	21,22,22,22	0
3	GOL	B	605	6/6	0.98	0.10	16,18,18,19	0
4	ZN	B	601	1/1	1.00	0.03	14,14,14,14	0

CONFIDENTIAL VALIDATION REPORT

The following is a graphical depiction of the model fit to experimental electron density of all instances of the Ligand of Interest. In addition, ligands with molecular weight > 250 and outliers as shown on the geometry validation Tables will also be included. Each fit is shown from different orientation to approximate a three-dimensional view.

Electron density around GOL A 605:

2mF$_o$-DF$_c$ (at 0.7 rmsd) in gray
mF$_o$-DF$_c$ (at 3 rmsd) in purple (negative)
and green (positive)

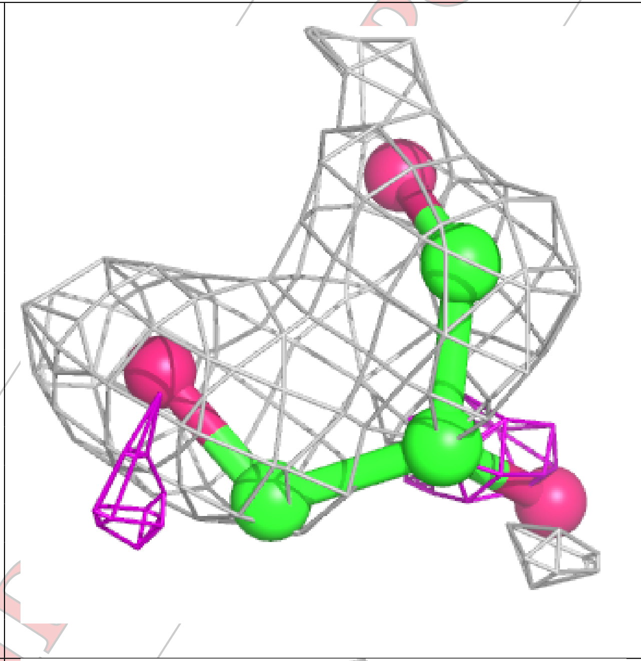

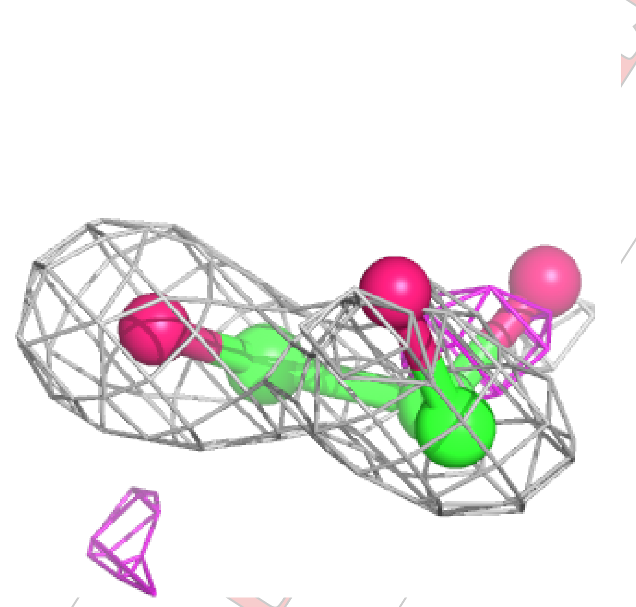

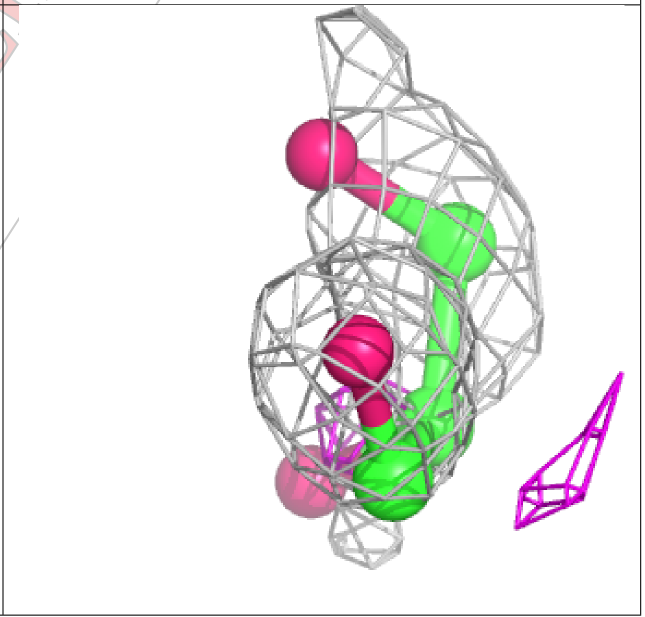

CONFIDENTIAL REPORT

Electron density around GOL A 607:

$2mF_o$-DF_c (at 0.7 rmsd) in gray
mF_o-DF_c (at 3 rmsd) in purple (negative)
and green (positive)

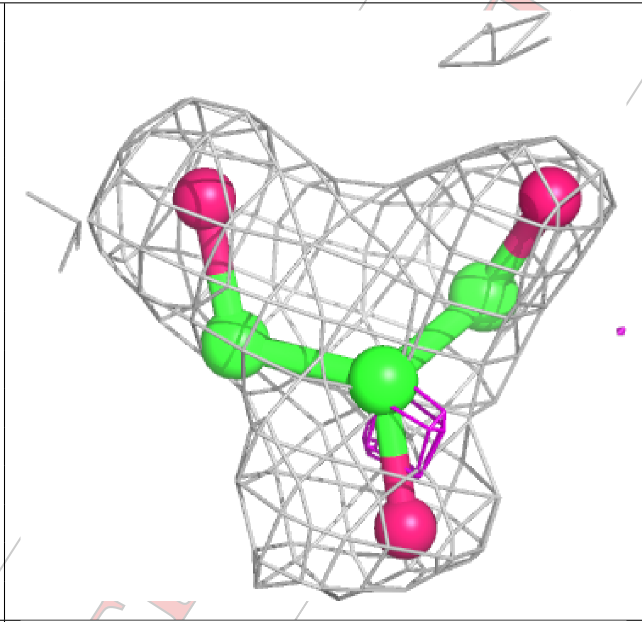

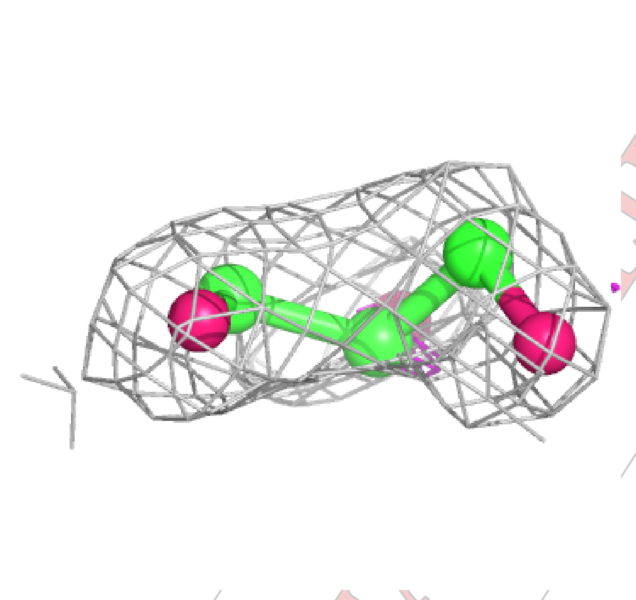

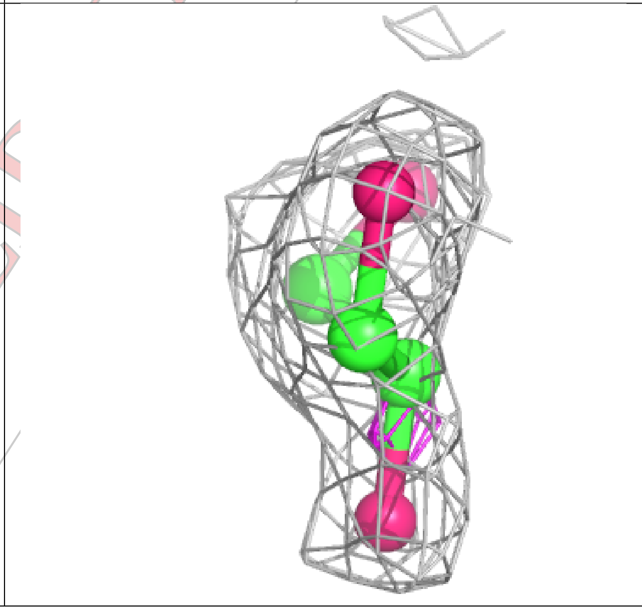

CONFIDENTIAL

Electron density around GOL B 607:

$2mF_o$-DF_c (at 0.7 rmsd) in gray
mF_o-DF_c (at 3 rmsd) in purple (negative)
and green (positive)

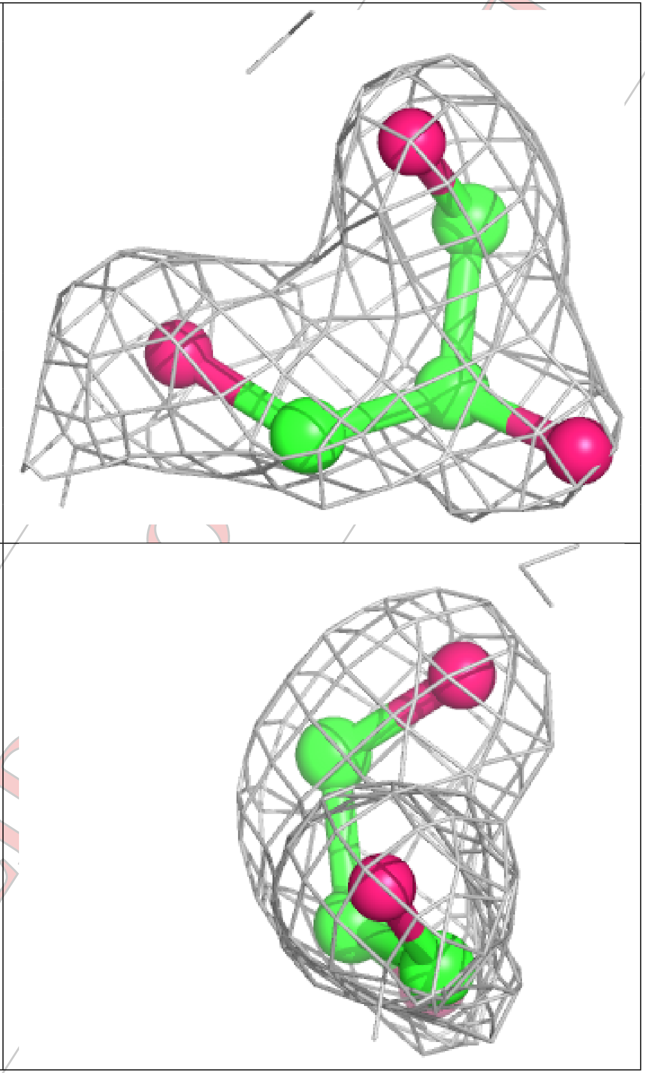

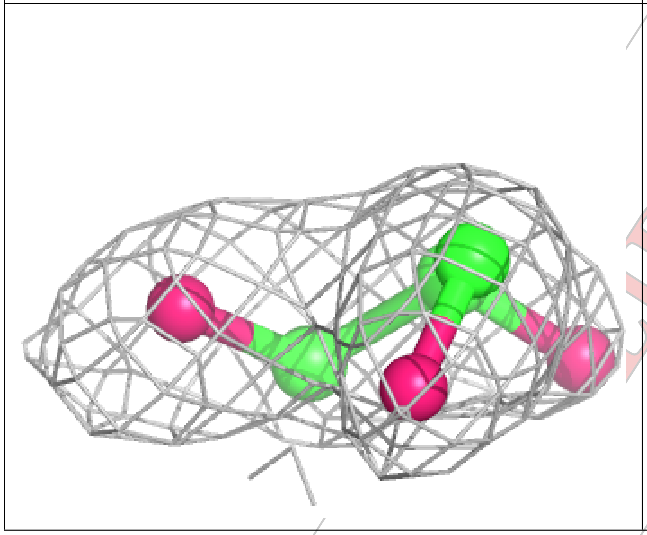

CONFIDENTIAL

Electron density around GOL A 604:

2mF$_o$-DF$_c$ (at 0.7 rmsd) in gray
mF$_o$-DF$_c$ (at 3 rmsd) in purple (negative)
and green (positive)

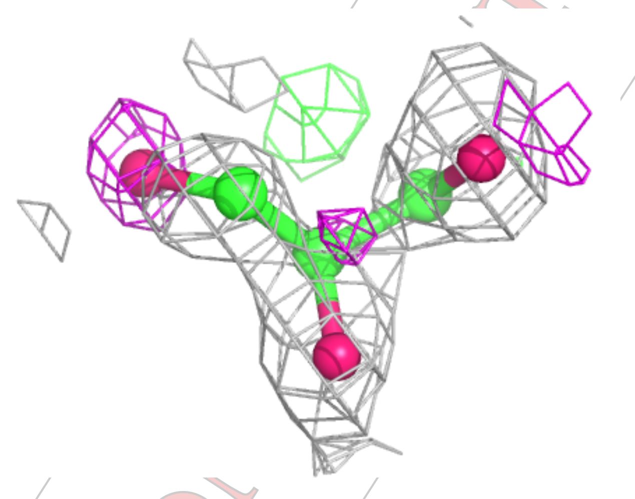

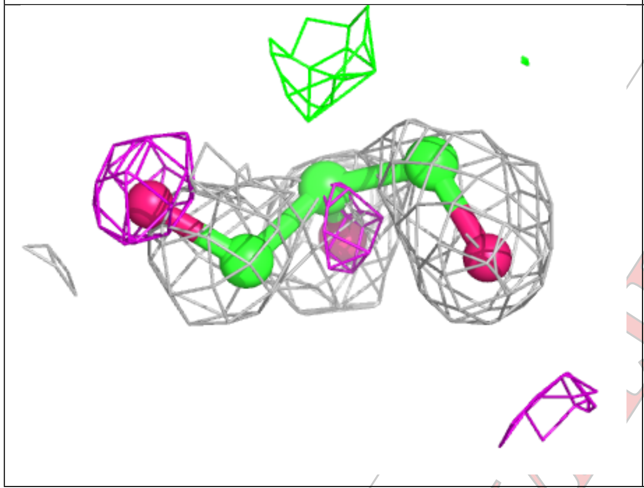

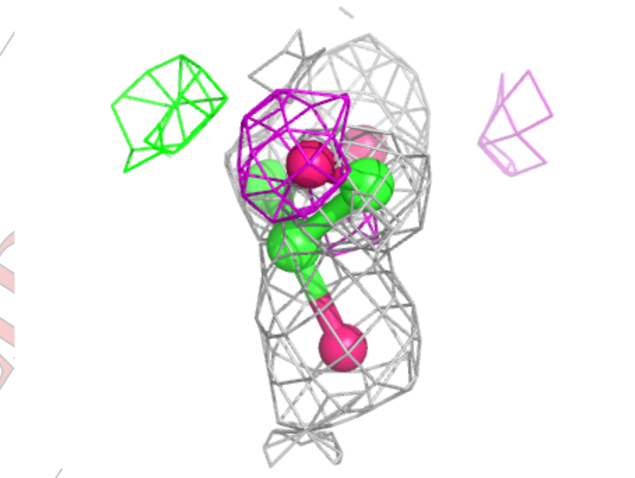

CONFIDENTIAL

Electron density around GOL B 606:

$2mF_o$-DF_c (at 0.7 rmsd) in gray
mF_o-DF_c (at 3 rmsd) in purple (negative)
and green (positive)

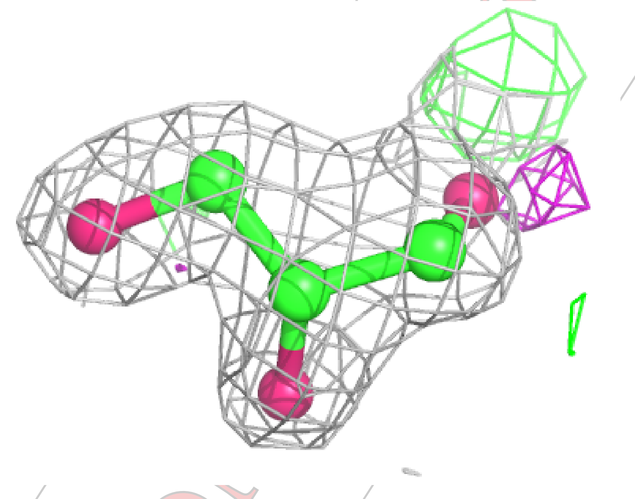

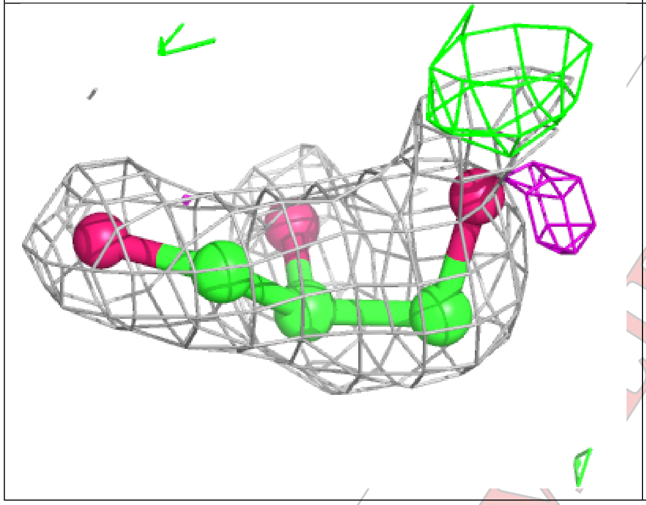

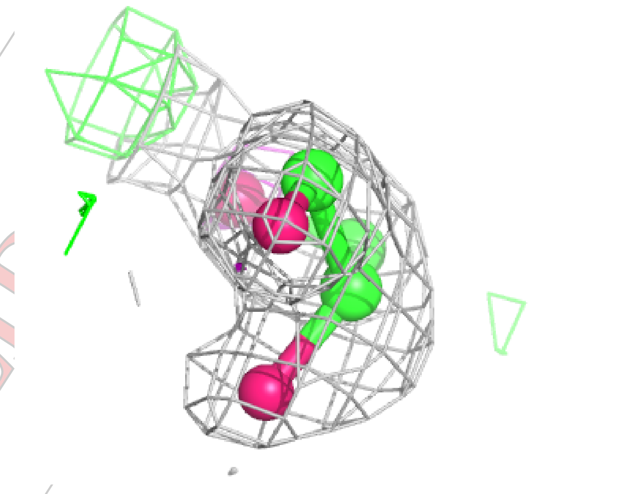

CONFIDENTIAL

Electron density around GOL A 603:

$2mF_o$-DF_c (at 0.7 rmsd) in gray
mF_o-DF_c (at 3 rmsd) in purple (negative)
and green (positive)

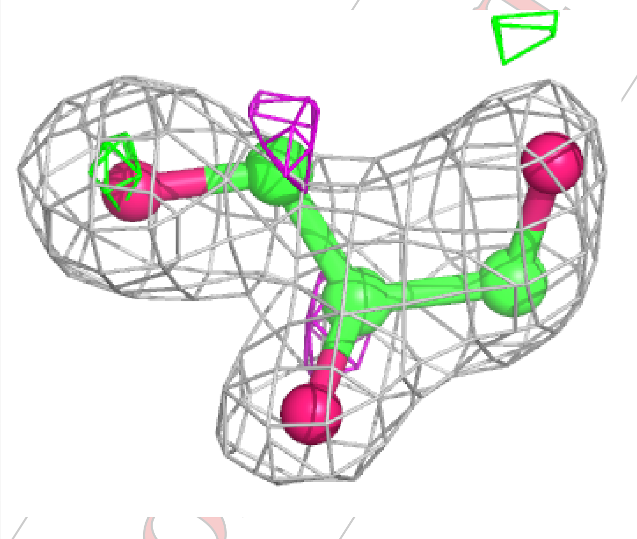

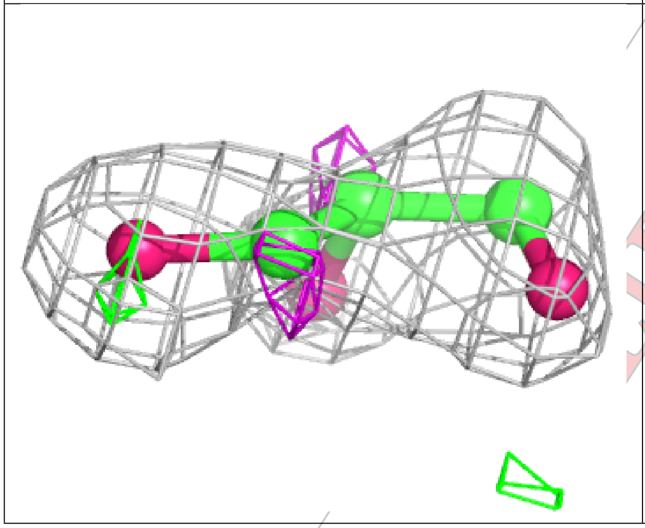

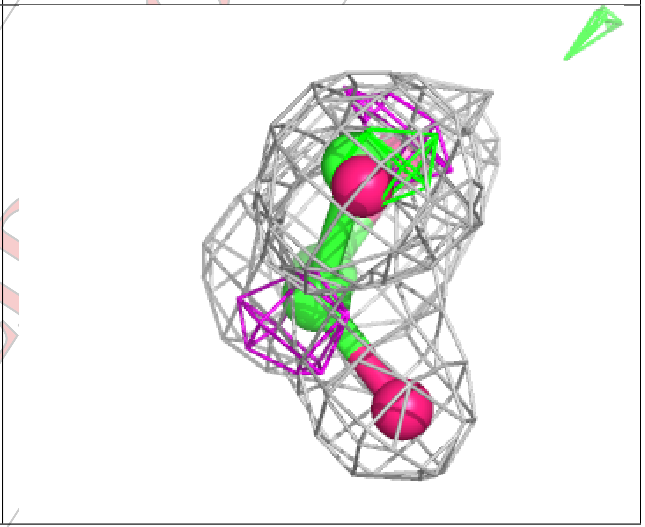

CONFIDENTIAL

Electron density around MES A 601:

2mF$_o$-DF$_c$ (at 0.7 rmsd) in gray
mF$_o$-DF$_c$ (at 3 rmsd) in purple (negative)
and green (positive)

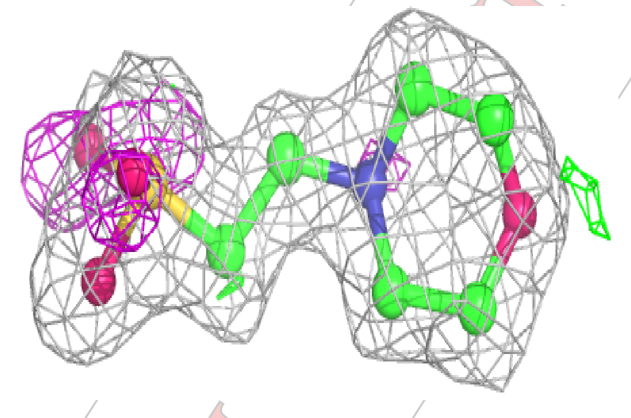

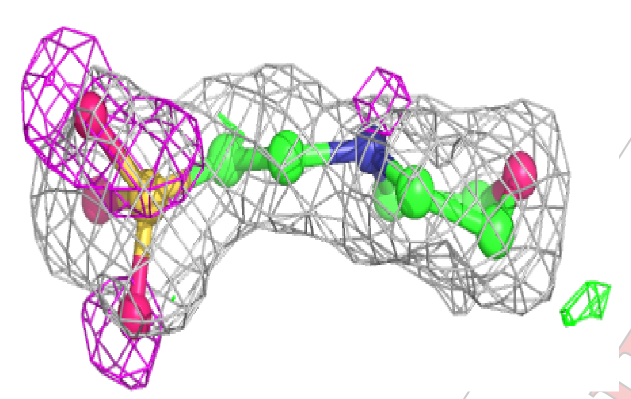

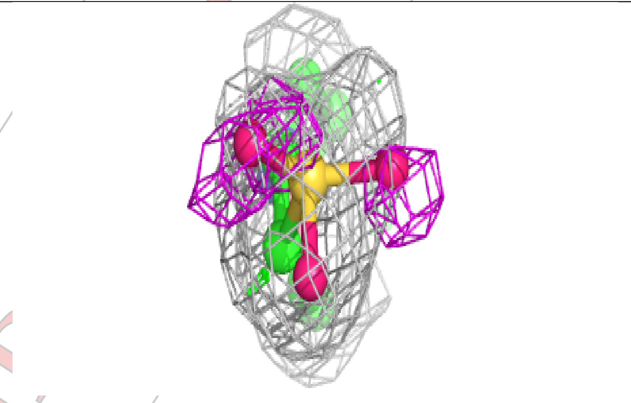

Electron density around GOL A 609:

$2mF_o$-DF_c (at 0.7 rmsd) in gray
mF_o-DF_c (at 3 rmsd) in purple (negative)
and green (positive)

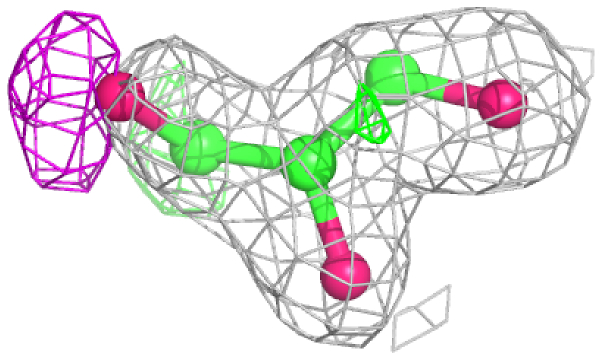

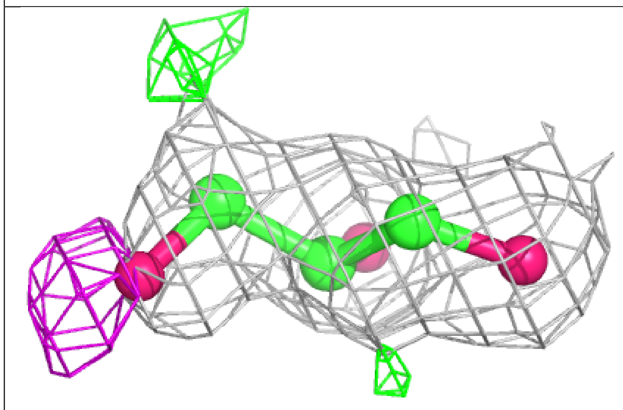

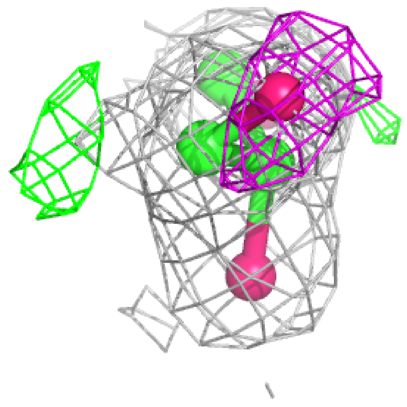

CONFIDENTIAL

Electron density around GOL A 606:

$2mF_o$-DF_c (at 0.7 rmsd) in gray
mF_o-DF_c (at 3 rmsd) in purple (negative)
and green (positive)

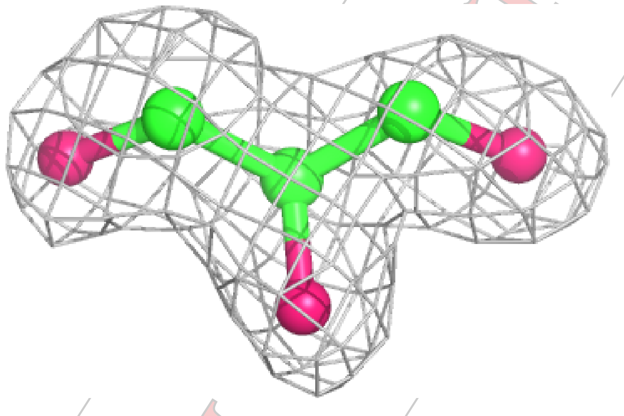

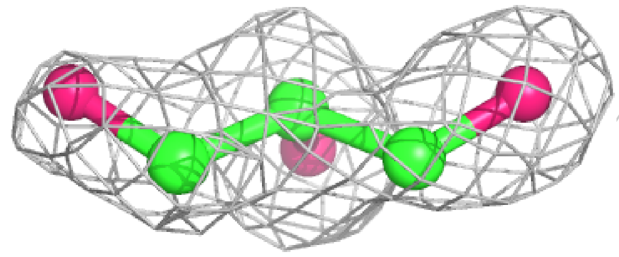

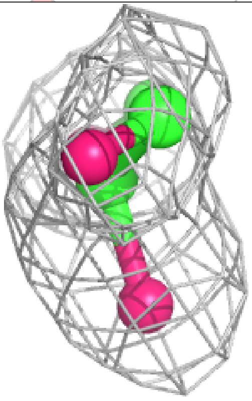

Electron density around MES B 602:

$2mF_o$-DF_c (at 0.7 rmsd) in gray
mF_o-DF_c (at 3 rmsd) in purple (negative)
and green (positive)

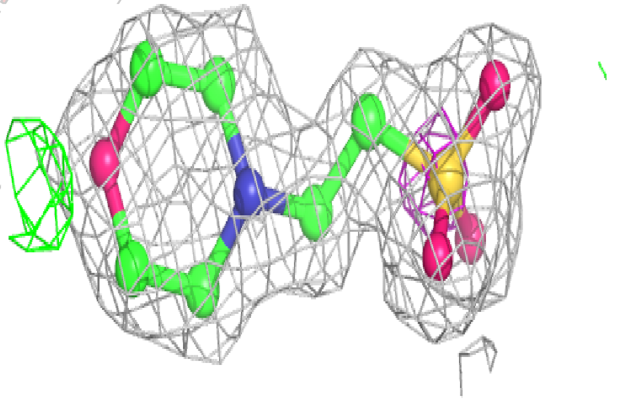

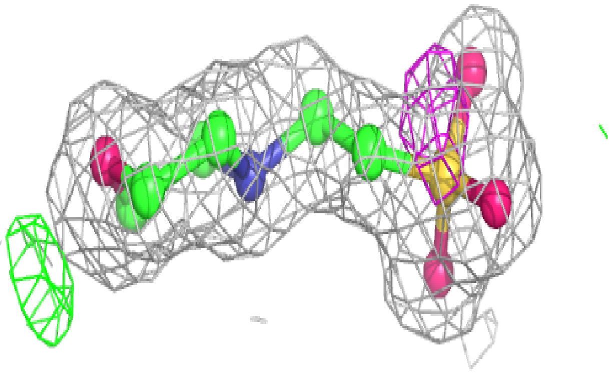

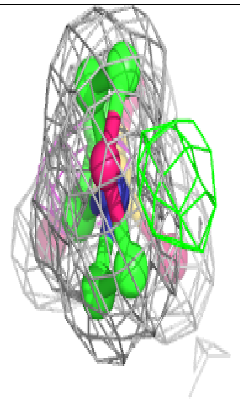

Electron density around GOL B 604:

2mF$_o$-DF$_c$ (at 0.7 rmsd) in gray
mF$_o$-DF$_c$ (at 3 rmsd) in purple (negative)
and green (positive)

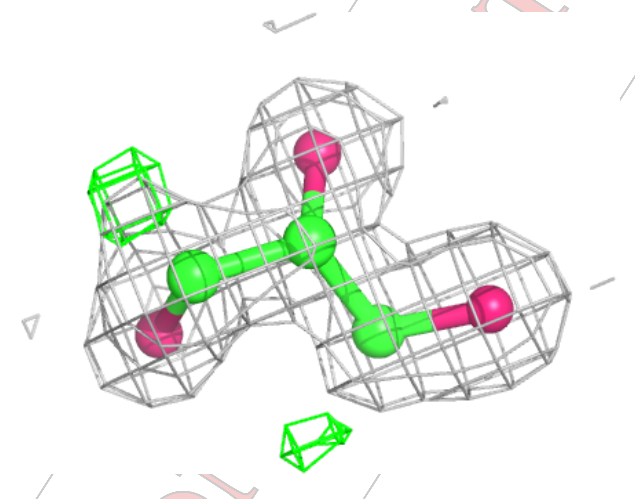

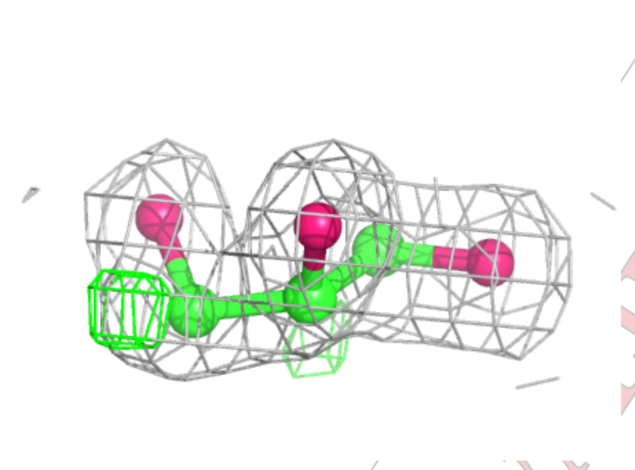

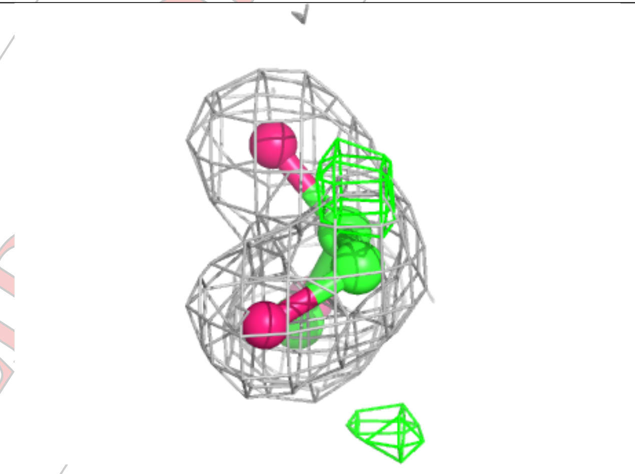

CONFIDENTIAL

Electron density around GOL A 602:

$2mF_o$-DF_c (at 0.7 rmsd) in gray
mF_o-DF_c (at 3 rmsd) in purple (negative)
and green (positive)

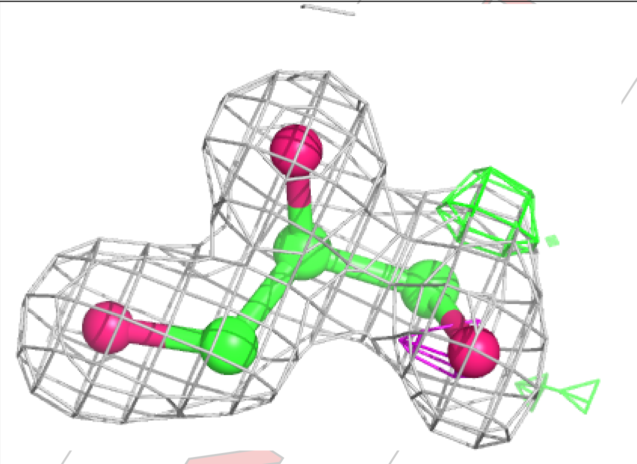

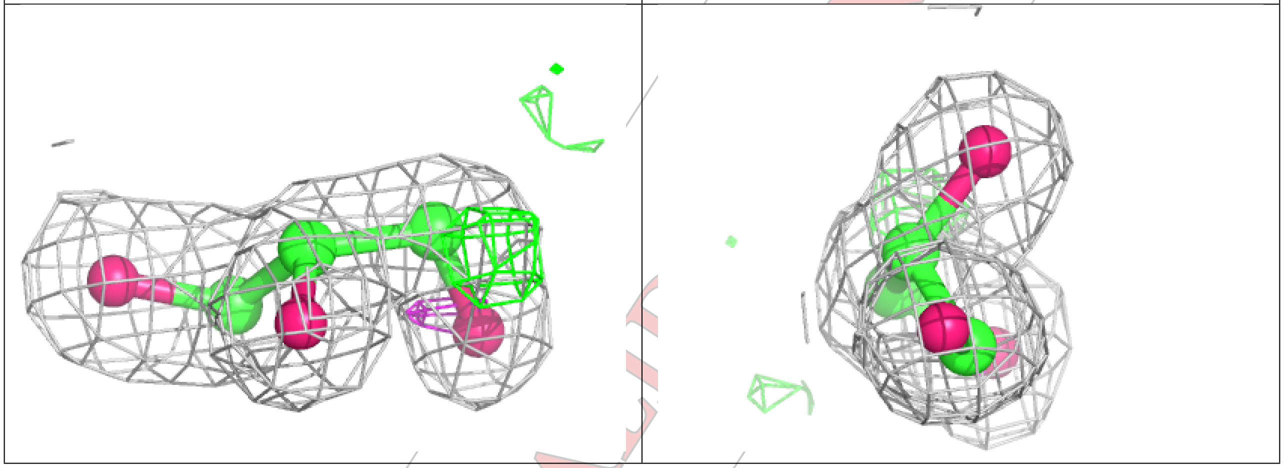

CONFIDENTIAL VA

Electron density around GOL A 608:

2mF$_o$-DF$_c$ (at 0.7 rmsd) in gray
mF$_o$-DF$_c$ (at 3 rmsd) in purple (negative)
and green (positive)

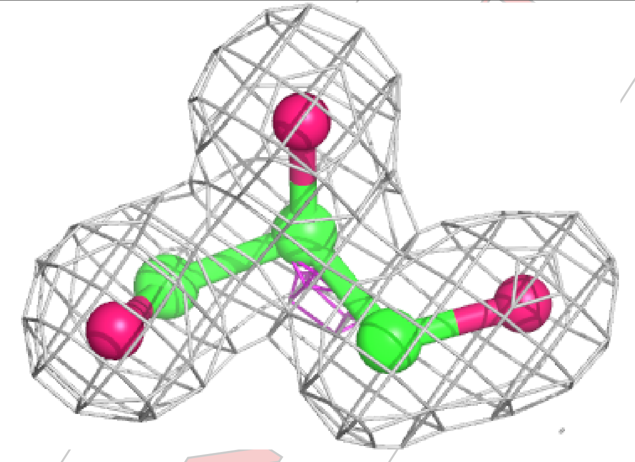

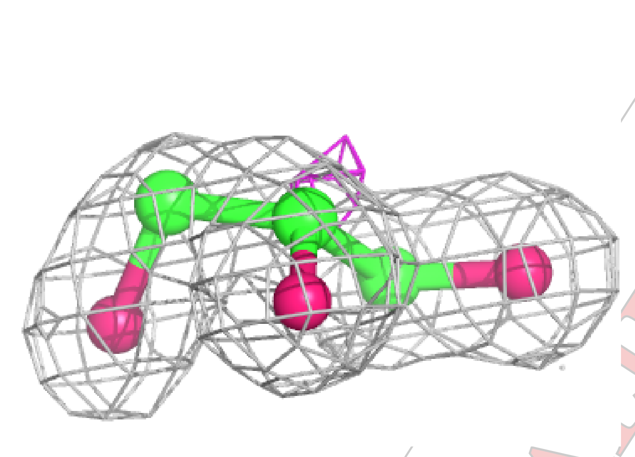

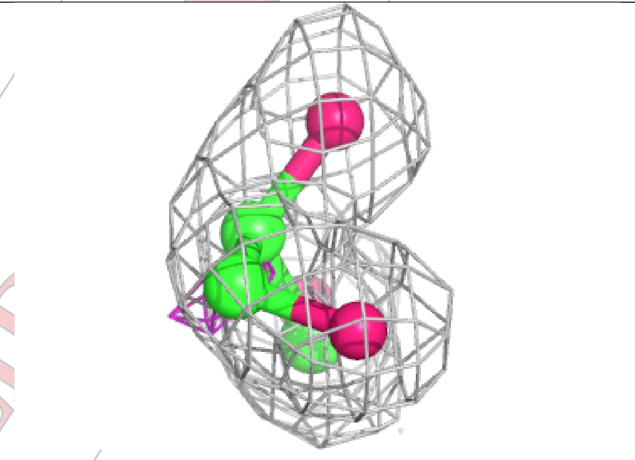

Electron density around GOL B 603:

2mF$_o$-DF$_c$ (at 0.7 rmsd) in gray
mF$_o$-DF$_c$ (at 3 rmsd) in purple (negative)
and green (positive)

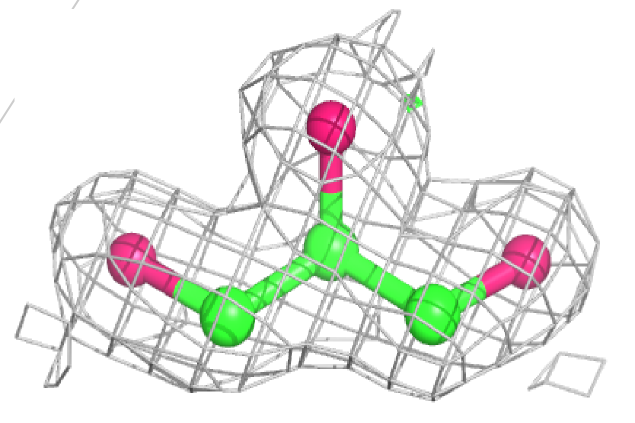

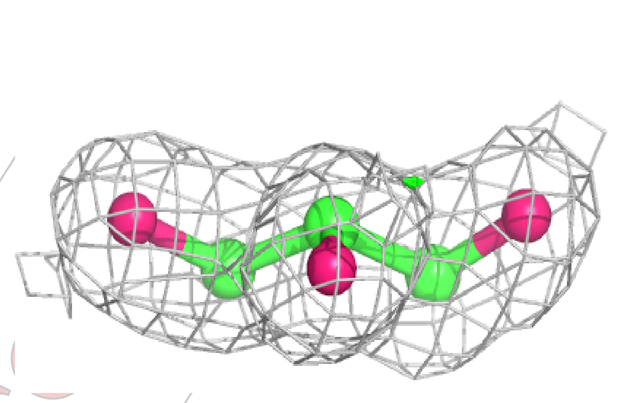

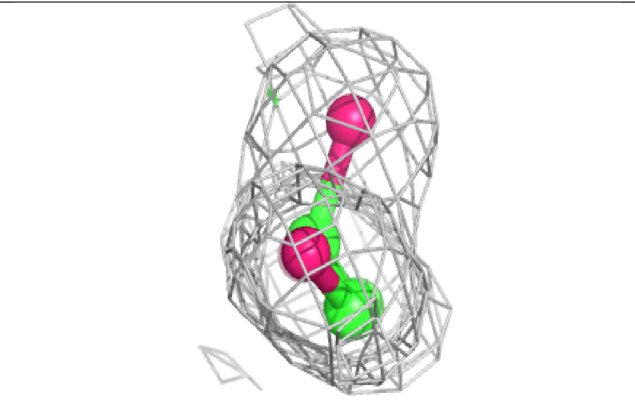

Electron density around GOL B 605:

2mF$_o$-DF$_c$ (at 0.7 rmsd) in gray
mF$_o$-DF$_c$ (at 3 rmsd) in purple (negative)
and green (positive)

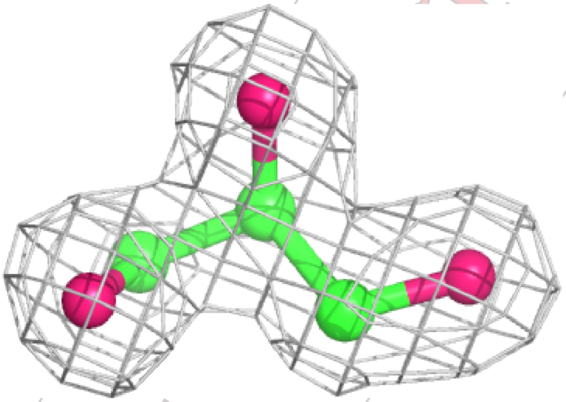

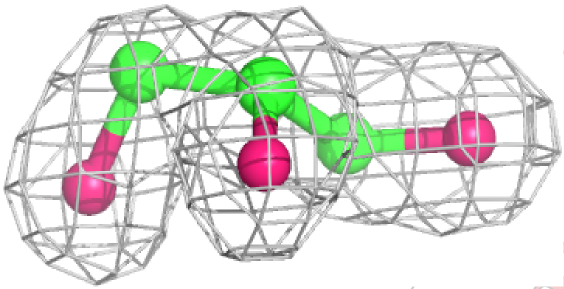

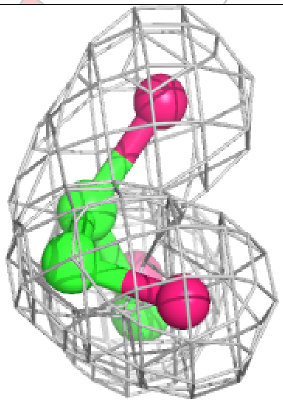

Electron density around ZN B 601:

$2mF_o$-DF_c (at 0.7 rmsd) in gray
mF_o-DF_c (at 3 rmsd) in purple (negative)
and green (positive)

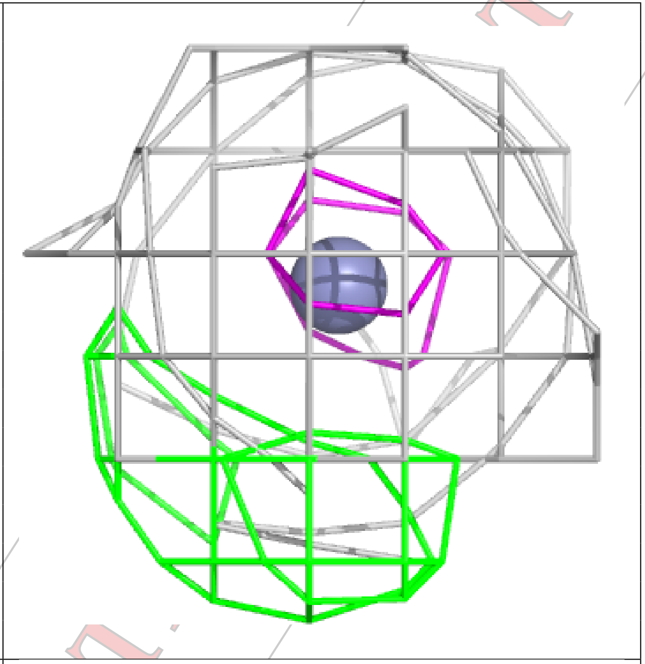

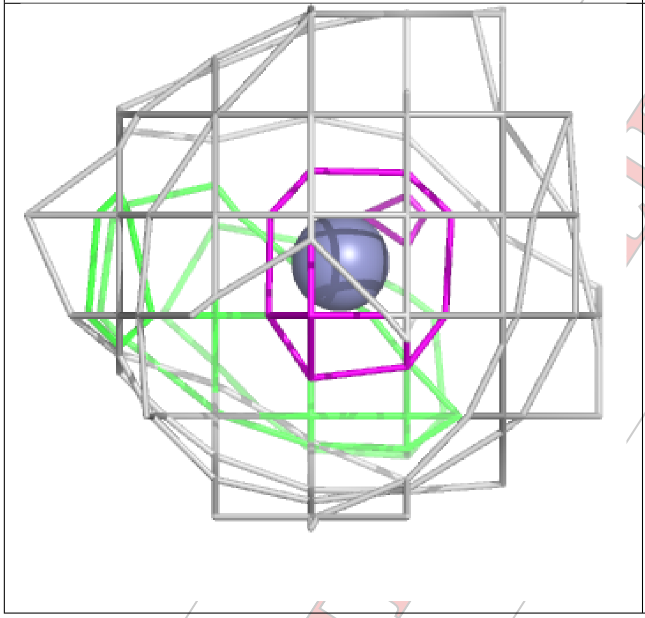

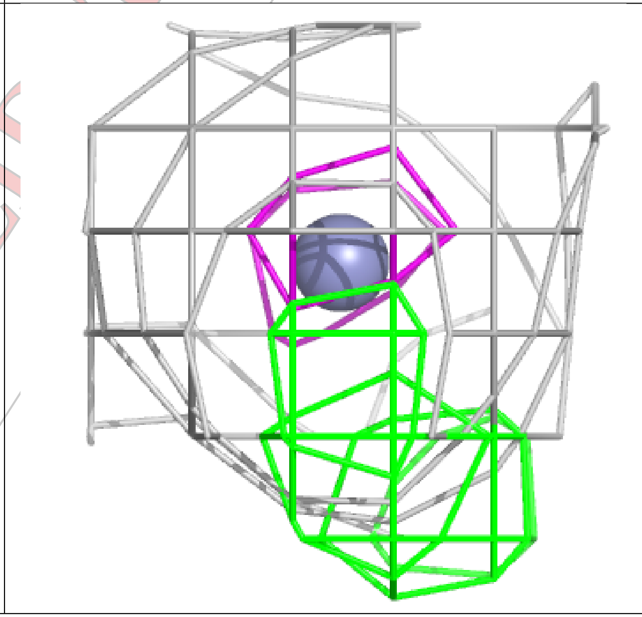

6.5 Other polymers ⓘ

There are no such residues in this entry.
